# Review and recommendations on deformable image registration uncertainties for radiotherapy applications

**DOI:** 10.1088/1361-6560/ad0d8a

**Published:** 2023-12-13

**Authors:** Lena Nenoff, Florian Amstutz, Martina Murr, Ben Archibald-Heeren, Marco Fusella, Mohammad Hussein, Wolfgang Lechner, Ye Zhang, Greg Sharp, Eliana Vasquez Osorio

**Affiliations:** 1 Department of Radiation Oncology, Massachusetts General Hospital, Boston, MA, United States of America; 2 Harvard Medical School, Boston, MA, United States of America; 3 OncoRay—National Center for Radiation Research in Oncology, Faculty of Medicine and University Hospital Carl Gustav Carus, Technische Universität Dresden, Helmholtz-Zentrum Dresden—Rossendorf, Dresden Germany; 4 Helmholtz-Zentrum Dresden—Rossendorf, Institute of Radiooncology—OncoRay, Dresden, Germany; 5 Department of Physics, ETH Zurich, Switzerland; 6 Center for Proton Therapy, Paul Scherrer Institute, Villigen PSI, Switzerland; 7 Division of Medical Radiation Physics and Department of Radiation Oncology, Inselspital, Bern University Hospital, and University of Bern, Bern, Switzerland; 8 Section for Biomedical Physics, Department of Radiation Oncology, University of Tübingen, Germany; 9 Icon Cancer Centres, Sydney, NSW, Australia; 10 Department of Radiation Oncology, Abano Terme Hospital, Italy; 11 Metrology for Medical Physics, National Physical Laboratory, Teddington, United Kingdom; 12 Department of Radiation Oncology, Medical University of Vienna, Austria; 13 Division of Cancer Sciences, The University of Manchester, Manchester, United Kingdom

**Keywords:** deformable image registration, DIR uncertainty, radiotherapy, strucutre propagation, dose accumulation, mapping, image deformation

## Abstract

Deformable image registration (DIR) is a versatile tool used in many applications in radiotherapy (RT). DIR algorithms have been implemented in many commercial treatment planning systems providing accessible and easy-to-use solutions. However, the geometric uncertainty of DIR can be large and difficult to quantify, resulting in barriers to clinical practice. Currently, there is no agreement in the RT community on how to quantify these uncertainties and determine thresholds that distinguish a good DIR result from a poor one. This review summarises the current literature on sources of DIR uncertainties and their impact on RT applications. Recommendations are provided on how to handle these uncertainties for patient-specific use, commissioning, and research. Recommendations are also provided for developers and vendors to help users to understand DIR uncertainties and make the application of DIR in RT safer and more reliable.

## 1. Introduction

Deformable image registration (DIR) is used in multiple applications in radiotherapy (RT), including image fusion, contour propagation, dose mapping, and dose accumulation. Many improvements in patient quality of care may be facilitated by DIR, including clinical delineations using multiple images (Brock *et al* 2017, Barber *et al* 2020), organ sparing with adaptive techniques (Albertini *et al* 2020, Glide-Hurst *et al* 2021), and better understanding of patient morbidity and mortality risks incorporating adaptive RT (ART) with accumulated dose (Murr *et al* 2023, Smolders *et al* 2023b). The efficacy of these techniques relies on the accuracy and reproducibility of the results of DIR. Incorporation of DIR-facilitated processes without an understanding of the impact of uncertainties may affect RT patient treatments.

The potential and risks of DIR in RT are well covered in current literature (Brock *et al* 2017, Paganelli *et al* 2018, Lowther *et al* 2022, Murr *et al* 2023). The American Association of Physicists in Medicine Task Group 132 (AAPM TG-132) report (Brock *et al* 2017) provided early guidance for work on qualification and commissioning of DIR algorithms and processes. AAPM TG-132 remains an excellent review of DIR and quality assurance (QA), but the report does suffer from some limitations. Latifi *et al* noted difficulties in applying the AAPM TG-132 recommendations in clinical practice (Latifi *et al* 2018) . Hussein *et al* and Rigaud *et al* report barriers to DIR clinical implementation with a lack of suitable evaluation tools and consensus on their implementation (Rigaud *et al* 2019, Hussein *et al* 2021). Barber *et al* and Paganelli *et al* addressed the requirements of patient-specific DIR QA and commissioning, and discussed the difficulties of consensus DIR QA metrics (Paganelli *et al* 2018, Barber *et al* 2020). Recent position papers out of the Australasian College of Physical Scientists and Engineers in Medicine (ACPSEM) (Barber *et al* 2020) and the Medical Image Registration Special Interest Group (MIRSIG) (Lowther *et al* 2022) have proposed consensus evaluation strategies for local geometric accuracy and vector grid suitability.

Despite recommendations on geometric tolerances present in the literature, the reporting of uncertainty quantification in clinically implemented DIR is not well standardised for RT applications in today’s literature, particularly with respect to dosimetric measures. This review aims to summarise the current understanding of uncertainties in DIR-facilitated processes and their clinical impact. The authors analysed the current literature about uncertainties in multiple DIR-facilitated applications, and summarised and extended recommendations with the general aim of raising awareness.

This review is structured as follows: We first summarise DIR algorithms used in RT (Chapter 2), and give a short explanation about the sources of DIR uncertainties (Chapter 3). Next, we review methods to quantify DIR uncertainties geometrically and dosimetrically (Chapter 4), and describe the effects and severity of these uncertainties for different RT applications (Chapter 5). Finally, we discuss uncertainty tolerances (Chapter 6) and summarise and expand current recommendations and recommend future research avenues (Chapter 7).

## 2. DIR algorithms

**Figure 1.**
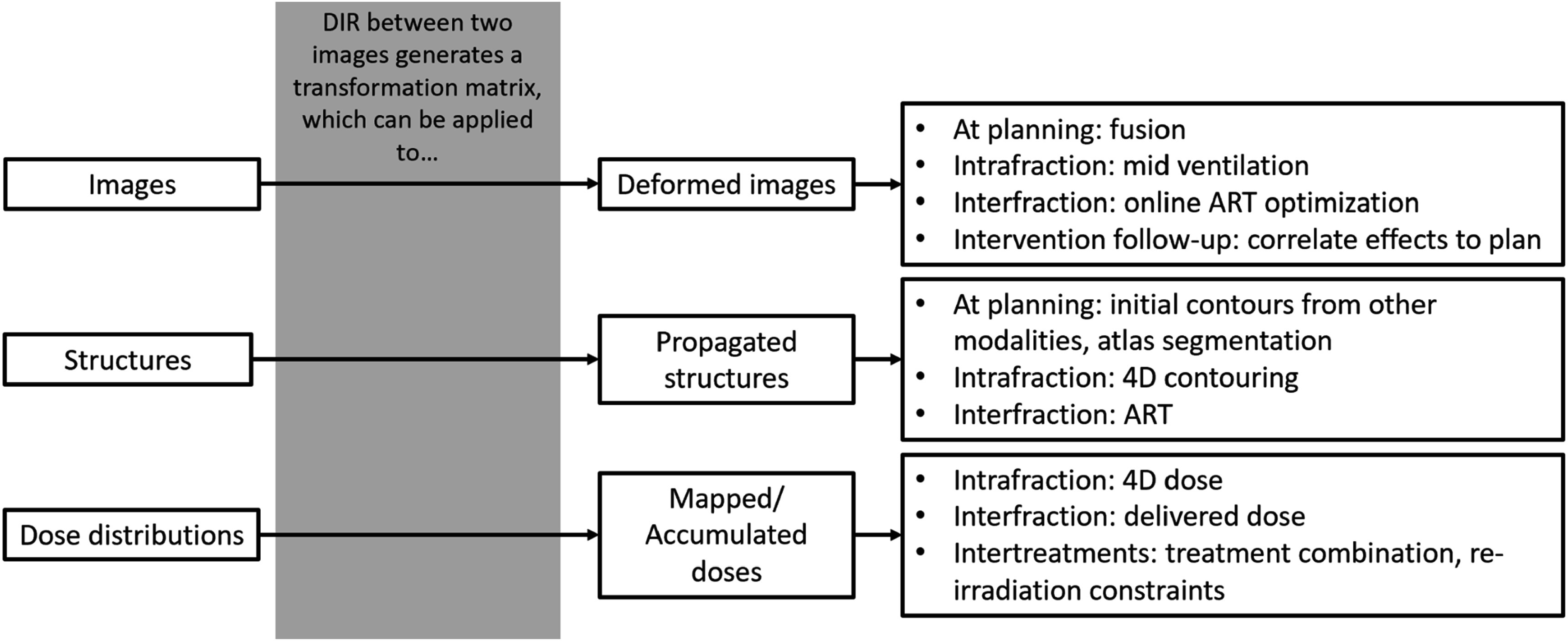
Schematic overview of radiotherapy applications influenced by uncertainty in DIR-generated transformations. DIR: deformable image registration, ART: adaptive radiotherapy.

DIR is applied between two images, aiming at aligning corresponding anatomic regions in both images. The result of a DIR is a transformation, which is often represented as a displacement vector field (DVF), which can be applied to images, structures, or dose distributions (figure 1). The earliest DIR algorithms were based on optical flow (Horn and Schunck 1981) or thin plate splines (Bookstein 1989). Classical algorithms, such as intensity-based matching or biomechanical models remain popular, but recently research in deep-learning (DL) methods is increasing. For a comprehensive overview of DIR algorithms, we refer the reader to review articles (Maintz and Viergever 1998, Holden 2008, Haskins *et al* 2020, Chen *et al* 2021, Teuwen *et al* 2022, Zou *et al* 2022).

### 2.1. Classical image registration

**Figure 2.**
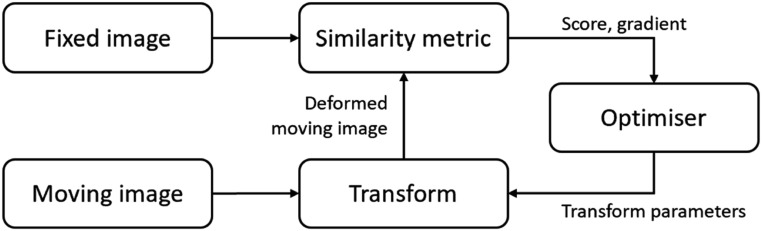
Classical image registration optimises transformation parameters by comparing a fixed image against a warped moving image. This figure is inspired by and adapted from the ITK Software Guide, reproduced with change under the Creative Commons Attribution 3.0 Unported License (Johnson *et al* 2019).

Classical methods, in their simplest form, follow a process illustrated in figure 2. There are two input images, a moving image and a fixed image, where the goal is to deform the moving image into the coordinate system of the fixed image. The algorithm proceeds by iteratively optimising transformation parameters to find a registration that minimises a similarity metric. The transformation parameters represent a displacement field, a velocity field, spline parameters, or other deformable transform representations. The similarity metric typically includes a regularisation term, which limits permissible transformations to those considered desirable or physically plausible, in addition to a similarity metric that matches intensity, such as mutual information or correlation coefficient.

Intensity-based DIR matching criteria are developed to use image intensity to optimise metrics such as mutual information (MI), sum of the squared difference (SSD) of image intensity, or cross-correlation (CC) (Oh and Kim 2017, Li *et al* 2021). Intensity-based DIR can achieve high accuracy for image areas with clear image features and high contrast. In poor contrast regions, intensity-based DIR accuracy may be less robust (Elmahdy *et al* 2019, Li *et al* 2021, Tascón-Vidarte *et al* 2022). To improve DIR accuracy, hybrid DIR algorithms consider point landmarks or structures defined on both image sets to improve registration results (Zhong *et al* 2012, Weistrand and Svensson 2015, Qin *et al* 2018, Motegi *et al* 2019, Shah *et al* 2021). Some algorithms rely on distance criteria to determine correspondence and transformations (Xiong *et al* 2006, Vásquez Osorio *et al* 2009, Zakariaee *et al* 2016) others use biomechanical properties.

Biomechanical algorithms are influenced by modelled physical properties of the tissues (Sotiras *et al* 2013, Polan *et al* 2017, Velec *et al* 2015, 2017). Finite element methods (FEM) model the properties of the tissues under mechanical force. Although the use of FEM requires the challenging definition of material properties, geometry, and boundary conditions, its robustness and plausibility are well demonstrated (Sotiras *et al* 2013). Compared to intensity-based DIR, it can improve multi-modal registration and registration in low-contrast regions (Velec *et al* 2015).

### 2.2. Deep learning-based DIR

In the past decade, machine learning algorithms in radiotherapy have increased dramatically, and DL has likewise made advances in the field of medical DIR (Teuwen *et al* 2022, Zou *et al* 2022). Topical reviews of the literature present extensive summaries of the current state of DL algorithms within DIR (Boveiri *et al* 2020, Xiao *et al* 2021, Zou *et al* 2022). DL in image registration is implemented through two approaches: deep similarity metrics in classical image registration algorithms, and deep neural networks that directly estimate the DVF.

#### 2.2.1. Deep similarity metrics (DSMs)

As described in section 2.1, classical algorithms approach the problem of image alignment through a process of iterative optimization. These algorithms search for a global minimum of the solution space, but the choice of similarity metric remains problematic. DSMs aim to improve classical iterative image registration by improving the similarity term. This approach is particularly useful in multi-modal imaging where it has been shown to outperform mutual information (Wu *et al* 2013, Simonovsky *et al* 2016). Improvements in difficult monomodal registration problems, low contrast regions and large transformations, have been reported in the literature (Zhao and Jia 2015).

#### 2.2.2. Direct determination of DVFs by machine learning algorithms

Direct DVF DL algorithms use historic DVFs or artificial DVFs as training data to determine registrations. The optimization phase happens in the training phase, where model parameters are determined. The vast majority of DL models aim for a direct regression of DVF transforms in a supervised approach. Variation between models is primarily a result of algorithm design and methodology.

Reviews (Boveiri *et al* 2020) cover a range of algorithm architectures. DL architectures include staked auto-encoders (SAEs) (Wang *et al* 2017, Krebs *et al* 2018), bayesian frameworks (Deshpande and Bhatt 2019, Khawaled and Freiman 2020, 2022a), implicit neural representations (Wolterink *et al* 2022) and convolution neural networks (CNNs) (Cao *et al* 2018, Ferrante *et al* 2018, Hu *et al* 2018, Balakrishnan *et al* 2018, 2019, Kim *et al* 2019, Kuang and Schmah 2019, Liu *et al* 2019, Jian *et al* 2022, Wolterink *et al* 2022, Xi *et al* 2022, Liang *et al* 2023). CNNs have been researched for direct DVF regression, with reported improvements in DVF when coupled with spatial transformer networks (Jaderberg *et al* 2015). CNN architecture use encoder-decoder networks, rather than a fully connected layer. Such approaches are currently implemented in well-cited solutions (VoxelMorph (Balakrishnan *et al* 2018, 2019) and U-NET (Liang *et al* 2023)). Despite the growth of multimodal foundational models in image creation, these reviews do not find application in image registration.

In general, DL training is divided between supervised and unsupervised learning methods (Chen *et al* 2021). For supervised registration methods, ground truth is either a DVF or a segmentation. The DVF may be created by a conventional DIR algorithm or from synthetic deformations, and the segmentations may be created by manual contouring or other methods. Unsupervised registration methods are further split into training by similarity metrics or generative adversarial networks (GANs) (Mahapatra *et al* 2018, Elmahdy *et al* 2019). If similarity metrics are used no ground truth is needed for the learning process but, as in traditional image registration, these models are limited by the same issues as similarity metrics in classical DIR optimization. If GAN is used, a discriminator judges if the warped moving image can be discriminated from the fixed image. When the warped image cannot be distinguished from the fixed image, the registration is deemed to be optimal (Goodfellow *et al* 2014). GANs show promise for multi-modality DIR problems as they do not require image similarity terms.

One advantage of DL algorithms is improvements in multi-modal registration, which is challenging for classical similarity metrics. Additionally, DL-based algorithms are more computationally efficient (Rohé *et al* 2017, Cao *et al* 2018, Balakrishnan *et al* 2018, 2019).

## 3. Source of uncertainties

The uncertainties of DIR can arise from a variety of sources. Many are image-based uncertainties, caused by anatomical changes, artifacts and different image modalities, as well as algorithm-based uncertainties, caused by intrinsic mathematical limitations and similarity metrics.

### 3.1. Image-based

#### 3.1.1. Anatomical changes

**Figure 3.**
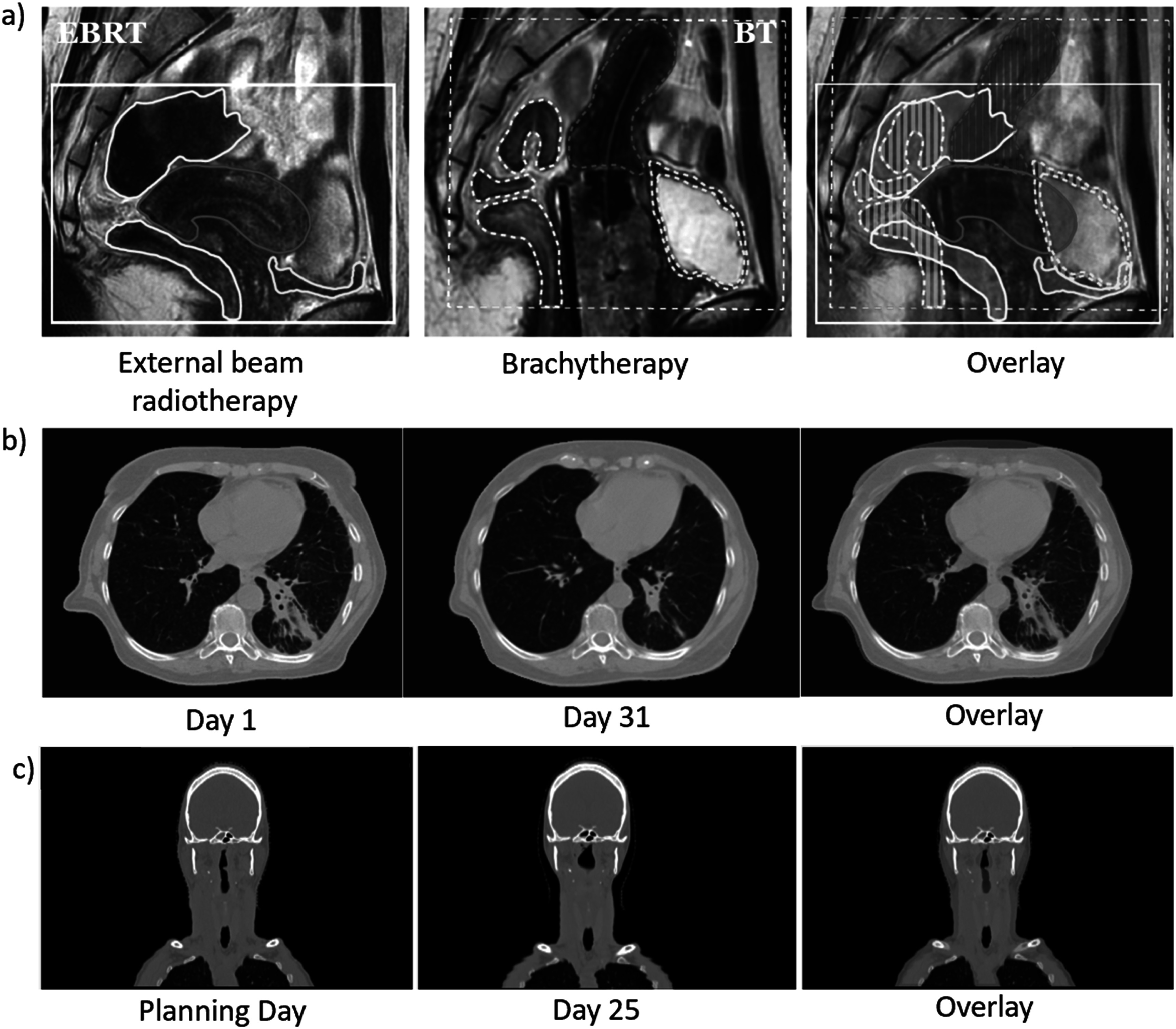
Examples of large anatomical changes. (a) Large changes during combined treatments with external beam radiotherapy (EBRT) and brachytherapy (BT) of the uterus. Image from (Vásquez Osorio *et al* 2015) with permission. (b) Large anatomical changes in the lung. (c) Weight loss for a head and neck patient during the course of treatment.

Non-rigid variations in patient anatomy, such as weight gain or loss, neck flexion and tumour changes can be poorly mapped by rigid and affine registrations. DIR can improve the locally accurate alignment of anatomy (Hill *et al* 2001). While regularisation is useful to reduce the likelihood of physically unrealistic deformations, the magnitude of anatomical changes may exceed those allowed by an algorithm’s settings. This can result in large DIR errors in areas near significant shape changes, particularly in low contrast image regions (Kashani *et al* 2008) or due to forced anatomical changes such as between external beam RT and brachytherapy (Vásquez Osorio *et al* 2015) (figure 3(a)).

Anatomical changes can be elastic, where the surrounding tissue follows the change and occupies the previous space (e.g. movement, position changes, or displacement) or inelastic, where the surrounding tissue stays in place (e.g. tissue growth, regression or emptying/filling cavities) (Amugongo *et al* 2022) figures 3(b) and (c). Modelling these changes is challenging (Sonke and Belderbos 2010, Mencarelli *et al* 2014, Sonke *et al* 2019). Certain implementations of regularisation can result in significant registration inaccuracies in sites in which naturally sliding boundaries occur, such as a rib bone and its adjacent lung (Sonke *et al* 2019). Some solutions were proposed to incorporate missing tissue during the DIR (Nithiananthan *et al* 2012, Vishnevskiy *et al* 2017, Eiben *et al* 2018).

#### 3.1.2. Artifacts/Image quality

**Figure 4.**
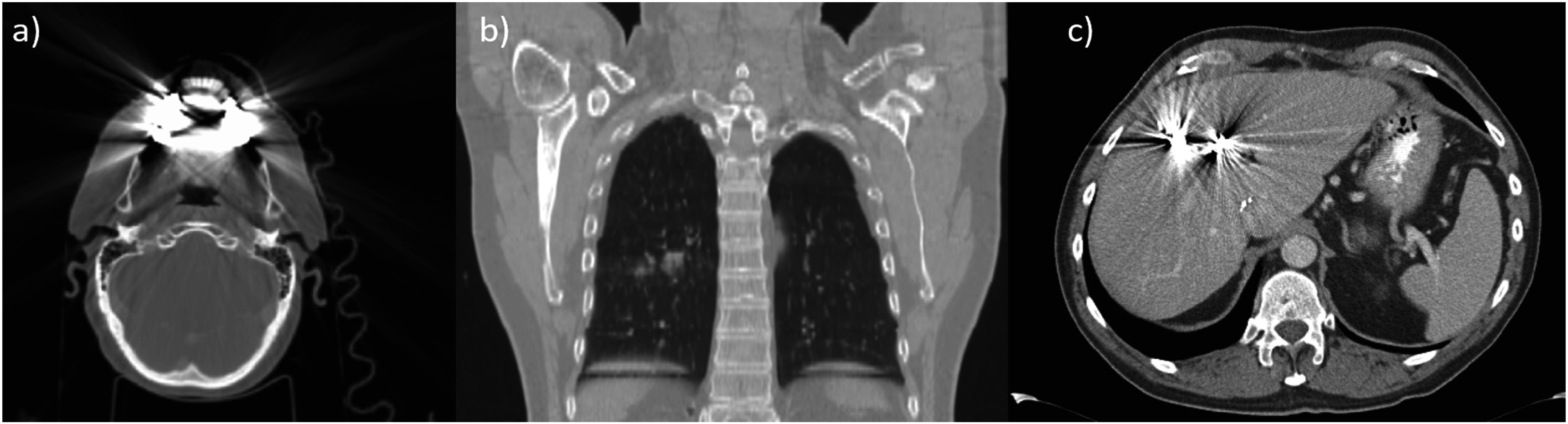
Examples of (a) dental artifacts (image from the United States National Cancer Institute (NCI) ‘The cancer imaging archive’ (TCIA) (Clark *et al* 2013, Ang *et al* 2014, Bosch *et al* 2015)), (b) 4 D artefacts in lung (image from TCIA (Roman *et al* 2012, Balik *et al* 2013, Clark *et al* 2013, Hugo *et al* 2017)) and (c) metal artifact in liver.

The anatomical changes caused by natural patient motion, such as respiration, muscle contraction, and blood flow can lead to image artifacts (Nehmeh and Erdi 2008, Zhang *et al* 2012, Spin-Neto and Wenzel 2016, Giganti *et al* 2022). For example, motion artifacts during the image acquisition can result in implausible anatomy (Yamamoto *et al* 2008, Persson *et al* 2010) and implants such as prostheses in the imaging area can lead to streaking or voids (Ritter *et al* 2009, Fontenele *et al* 2018, Lee *et al* 2021). As these artifacts disrupt the true image intensity gradients of the patient tissue several papers have demonstrated decreased intensity-based DIR quality in their presence (Serban *et al* 2008, Sonke and Belderbos 2010, Fusella *et al* 2016) (figure 4).

Sensitivity of DIR algorithms to image noise, resolution (Constable and Henkelman 1991, Verdun *et al* 2015, Zhao *et al* 2016, Sarrut *et al* 2017), field of view (Barber *et al* 2020) and image contrast (Mencarelli *et al* 2014, Barber *et al* 2020, Dowling and O’Connor 2020) has been demonstrated in the literature. However, other studies find that the effect of image noise has only minor effects on DIR results for computed tomography (CT) to CT registrations (Nesteruk *et al* 2022).

Research on the implementation of iterative image reconstruction algorithms has shown reduced noise and improved image quality for both CT and cone-beam CT (CBCT) (Held *et al* 2016, Giacometti *et al* 2019, Jarema and Aland 2019, Greffier *et al* 2020, Loi *et al* 2020), which may allow for improved quality intensity-based DIR.

#### 3.1.3. Multimodal registration

Multimodal DIR offers considerable clinical benefit in contour propagation (Söhn *et al* 2008, Vásquez Osorio *et al* 2012, Barber *et al* 2020, Zachiu *et al* 2020). However, multimodal DIR remains challenging, and similarity metrics must be selected with care.

**Figure 5.**
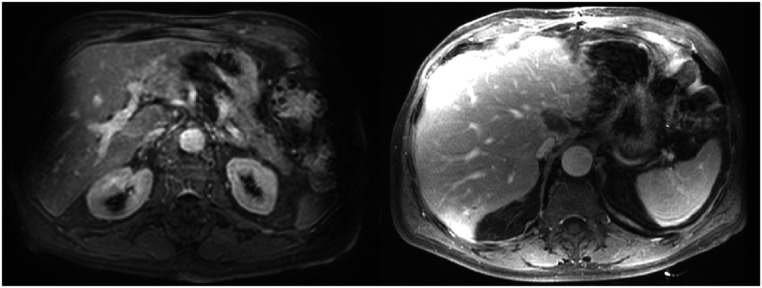
Example of gradient effects in MRI that may increase DIR uncertainties. Figure from (Vásquez Osorio *et al* 2012), with permission. MRI: magnetic resonance imaging, DIR: Deformable image registration.

For example, magnetic resonance imaging (MRI) to CT registration in the lung is difficult because of low contrast and resolution in MRI (Yang *et al* 2015) and in the prostate, lack of a clear boundary of the prostate gland in CT may lead to failures in MR-CT DIR (Zhong *et al* 2015). In the HN, limited soft tissue contrast and dental artifacts in CT images compared to MR influence the DIR uncertainty (Nix *et al* 2017, Kiser *et al* 2019). Additionally, gradient contrast artifacts in MRI may impair the DIR quality between different image modalities (figure 5) (Vásquez Osorio *et al* 2012). McKenzie at el. found monomodal registration from synthetic CT (generated from the MRI) to CT to be more accurate than the multimodal registration from the original MRI to CT for large deformations of HN patients (McKenzie *et al* 2020). Of course, the synthetic CT generation also faces uncertainties, for example the resulting Hounsfield units (HUs) differ between CT and synthetic CT. Boulanger *et al* report a mean absolute error of 76 HU in head and liver, and 42 HU in the pelvic area in average over multiple methods generating synthetic CTs (Boulanger *et al* 2021). Geometric differences between structures can also appear (Palmér *et al* 2021).

### 3.2. Algorithm-based

The choice of the DIR algorithm and parameter settings influences the DVF obtained when registering the same image pair. Several studies investigate the performance of different DIR algorithms, for example in HN (Hardcastle *et al* 2012, Močnik *et al* 2018, Qin *et al* 2018, Lee *et al* 2020, Kubli *et al* 2021), lung (Kadoya *et al* 2014, Scaggion *et al* 2020a), liver (Zhang *et al* 2012, Sen *et al* 2020) or pelvis (Hammers *et al* 2020). Some commercial DIR algorithms offer the possibility of parameter adjustments, such as registration metrics, guiding structures, regularisation levels, regularisation weights, or contrast level sensitivity, which causes uncertainty of the algorithm to vary (Ziegler *et al* 2019). However, most commercial algorithms are closed systems and not adjustable. Some studies find that even a single commercial DIR software can show variability in the results (Kadoya *et al* 2016, Miura *et al* 2017), depending on the specific workflows used. The performance of the same DIR algorithm might also vary between anatomical sites. For example, in a series of three separate studies comparing the Velocity and MIM algorithms (Kadoya *et al* 2016, Pukala *et al* 2016, Fukumitsu *et al* 2017) on different patient anatomies, the published results come to different conclusions regarding the performance.

## 4. Quantification of uncertainties

The quantification and evaluation of uncertainties in the applications of DIR are difficult due to multiple aspects. Firstly, a true ground truth is lacking and secondly, there are a wide range of DIR-facilitated applications which have differing requirements for accuracy. For dose monitoring, a low point-to-point error is necessary in steep dose gradients, while in low gradient or homogeneous dose regions, even larger point-to-point errors will not impact the mapped dose. For contour propagation, a high correspondence between organ boundaries is of importance (Rigaud *et al* 2019). Quantifying DIR uncertainties is crucial, as the DIR results are used for consecutive steps (Brock *et al* 2017, Paganelli *et al* 2018). So far, there is no standard procedure for uncertainty quantification of DIRs. Indeed, most commercial and research systems omit uncertainties entirely.

### 4.1. Using a digital or physical phantom as ground truth

The validation of DIR results is challenging due to the lack of ground truth. Therefore, evaluation strategies have been developed, questioned, and improved over the past decades. To create a ground-truth surrogate, digital phantoms and physical phantoms have been proposed. Digital phantoms (Castillo *et al* 2009, Vandemeulebroucke *et al* 2011, Brock *et al* 2017) are created using voxel-based reference deformations, which DIR algorithms are expected to recover. This can cause bias in results. For example, a digital phantom deformed with displacements generated by a B-spline might result in better agreement when testing algorithms that use B-spline transformations (Fatyga *et al* 2015, Loi *et al* 2018, Balakrishnan *et al* 2019, Boyd *et al* 2021). Digital phantoms allow for the comparison of contour-based evaluation methods *and* direct evaluation of DVF errors. In contrast, physical phantoms (Graves *et al* 2015, Niebuhr *et al* 2019, Kadoya *et al* 2021) provide geometrical verification through landmarks or structures. Therefore, physical phantoms suffer from a similar lack of ground truth as patient images. Intrinsic errors due to inter- and intra-observer variability due to the manual identification (Machiels *et al* 2019, Roach *et al* 2019) remain present in phantoms. The use of markers (Machiels *et al* 2019), guidelines (Lin *et al* 2020), auto segmentation (Rey *et al* 2002, Yang *et al* 2018, Cardenas *et al* 2019, Schipaanboord *et al* 2019, Vrtovec *et al* 2020, Harrison *et al* 2022) and automated landmark extraction (Paganelli *et al* 2018) can reduce observer uncertainties, but are not necessarily more accurate. Also, just as with patient images, these methods quantify DIR performance only near the points or structures under consideration (Shi *et al* 2021) and do not provide a holistic assessment of the DIR performance. The deformations of physical phantoms might not always be anatomically realistic. While both, digital and physical phantoms, are useful for commissioning and QA of applications involving DIR, it is important to keep their weaknesses in mind.

### 4.2. Geometric and dosimetric uncertainty quantification

With the lack of ground truth, alternative measures have to be used to quantify the effects of DIR uncertainty. Most commonly geometric measures are used, comparing warped points of interest or structures to reference points and structures. These reference-based geometric measures are however not always available and have their own uncertainties, such as intra- and inter-observer variability. Reference-free measures have also been proposed, they can be applied without reference data. A short summary of various geometric uncertainty measures is given in table 1. For a more detailed overview about geometric measures and which methods are proposed for specific applications please refer to the AAPM TG 132 (Brock *et al* 2017) and MIRSIG (Lowther *et al* 2022). In addition to geometric measures multiple methods to visualise and quantify dosimetric uncertainties have been proposed (table 1).

**Table 1.** Description, strengths and limitations of commonly used geometric and dosimetric uncertainty quantification metrics. DVH: Dose-volume-histogram.

	Metric	Description	Strengths (+) /Limitations (−)
Reference- based	Target registration error (TRE)	• Distance between anatomical landmarks defined by different methods, e.g. warped with DIR versus physician-drawn reference (Fitzpatrick *et al* 1998, Datteri and Dawant 2012, Brock *et al* 2017)	+ Distance, in mm
		${\mathrm{\bullet }}\,{TRE}=\left|T\left({p}_{f}\right)-{p}_{m}\right|$	+ Spatially resolved
		${\mathrm{\bullet }}\,T\left({p}_{f}\right):$ estimated transformation of point from fixed image, ${p}_{m}$ position of point on moving image	− Reference points required (manual or automatic definition), additional inherent uncertainties, and time consuming definition
			− Validity depends point quantity and relevance
			− Limited to areas with sufficient image contrast
			− Requires reference/ground truth
	Dice similarity coefficient (DSC)	• Measure of the overlap between two contours (Dice 1945, Brock *et al* 2017)	+ Widely used, useful to compare to literature
		${\mathrm{\bullet }}\,{DSC}=\frac{2{|X}\bigcap {Y|}}{{|X|}+{|Y|}}{|X}\bigcap {Y|}:$ volume covered by both structures, ${|X|}+{|Y|}:$ volume covered by at least one of the structures	− Strongly volume dependent, lacks sensitivity for large structures
			− Special care needed for tubular structures
			− Hard to interpret/no meaningful unit
			− Requires reference/ground truth
	Hausdorff distance (HD)	• Maximum distance of the closest approach of each point on one contour to all points of the other contour (Hausdorff 1920, Huttenlocher *et al* 1993)	+ Distance, in mm
		${\mathrm{\bullet }}\,{HD}(X,Y)=\max (d(X,Y),d(Y,X))$ with $d(X,Y)={\max }_{{x}\in X}{\min }_{y\in Y}{||x}-{y||}$ $d\left(X,Y\right)$ distance between two pointsets	− Sensitive to outliers
			− Requires reference/ground truth
	Mean distance to agreement (MDA)	• Mean distance of the closest approach of each point on one contour to all points of the other contour (Vrtovec *et al* 2020)	+ Distance, in mm
		${\mathrm{\bullet }}\,{HD}(X,Y)={mean}(d(X,Y),d(Y,X))$	+ Less sensitive to outliers than HD
			− Misses local uncertainties
			− Requires reference/ground truth
	Centre of mass displacement (COM)	• Shift in center of mass between two structures (Choi *et al* 2011, Takayama *et al* 2017)	+ Distance, in mm
		${\mathrm{\bullet }}\,{COM}=\sqrt{{\mathrm{\Delta }}{x}^{2}+{\mathrm{\Delta }}{y}^{2}+{\mathrm{\Delta }}{z}^{2}}$ with ${\mathrm{\Delta }}{x}^{2}=\vec{{R}_{1,x}}-\vec{{R}_{2,x}},{\mathrm{\Delta }}{y}^{2}=\vec{{R}_{1,y}}-\vec{{R}_{2,y}},{\mathrm{\Delta }}{z}^{2}=\vec{{R}_{1,z}}-\vec{{R}_{2,z}}$ and $\vec{R}=\frac{1}{M}\int \int \int \rho \left(\vec{r}\right)\,\vec{r}{dV}$ M: mass of the structure, $\rho \left(\vec{r}\right)$ density distribution structure	− Lacks sensitivity to variations in contour boundary
			− Requires reference/ground truth
Reference- free measures	Distance discordance metric (DDM)	• Mean distance of points from moving images which are registered to the same point in the a fixed reference image (Saleh *et al* 2014)	+ Useful in contrast-poor areas
		• For mathematical description please refer to the original publication (Saleh *et al* 2014)	+ Spatially resolved, reference-free
			− Needs at least four registered images
	Local uncertainty metric (LU)	• Uncertainties within a uniformly-dense structures can be calculated based on points defined on the organ edges (Takemura *et al* 2018)	+ Spatially resolved, reference-free
		• For mathematical description please refer to the original publication (Takemura *et al* 2018)	+ Works in uniformly-dense regions
			− Requires contours
	Jacobian determinant	• The first derivative of the DVF, distinguish between regions which are locally expanding in volume J>1 and those shrinking with volume J<1 (Chung *et al* 2001)	+ Local volume gain/loss detection
		${\mathrm{\bullet }}\,J=\det \left(\frac{dT}{d\overrightarrow{x}}\right)=\det \left(\frac{d{T}_{x}}{{dx}}\,\frac{d{T}_{x}}{{dy}}\,\frac{d{T}_{x}}{{dz}}\,\frac{d{T}_{y}}{{dx}}\,\frac{d{T}_{y}}{{dy}}\,\frac{d{T}_{y}}{{dz}}\,\frac{d{T}_{z}}{{dx}}\,\frac{d{T}_{z}}{{dy}}\,\frac{d{T}_{z}}{{dz}}\,\right)$ with T the transformation	+ Spatially resolved, reference-free
			− Misleading for actual mass change
			− Necessary but not sufficient
	Harmonic energy (HE)	• A measure of the nonlinearity of the transformation, inversely proportional to the smoothness of the deformation (Forsberg *et al* 2012, Varadhan *et al* 2013)	+ Measure for smoothness
		${\mathrm{\bullet }}\,{HE}={\left|\left|{Jac}\right|\right|}_{F}=\sqrt{{{\sum }_{i=1}^{3}{\sum }_{j=1}^{3}\left|{t}_{{ij}}\right|}^{2}}$ beeing the Frobenius norm of the Jacobian	+ Spatially resolved, reference-free
			− Hard to interpret
			− Fails with sliding surfaces
	Inverse consistency error (ICE) / Transitivity error (TE)	• Applying a registration from image A to image B and then back to image A, it is assumed that all points will be mapped on their original position. ICE is defined as the difference between the original point and the transformed point mapped back to the fixed image grid (Bender and Tomé 2009), TE extends this idea to more than two images (Bender *et al* 2012)	+ Related to algorithm repeatability
			+ Spatially resolved, reference-free
			− No indication of accuracy in the result
			− Necessary but not sufficient
Dosimetric measures	Dose parameter variations and DVH bands	• Report of relevant dosimetric point variations (e.g. V95%, D2%, V10Gy, mean dose) and DVH bands caused by uncertainties in propagated structures or dose mapping/accumulation (Nassef *et al* 2016, Lowther *et al* 2020a, 2020b, García-Alvarez *et al* 2022)	+ Clinically relevant dosimetric parameters
		• A known or estimated DIR uncertainty is necessary, either simulated (Wang *et al* 2018, Smolders *et al* 2022b), DIR variations (Nenoff *et al* 2020, Amstutz *et al* 2021b) or with known reference deformations (Kirby *et al* 2016, Covele *et al* 2021)	+ Applicable for illustrating uncertainties caused by propagated structures and/or mapped/accumulated doses
			+ No reference required
			− Previous measure for DIR uncertainty is necessary
	Local uncertainty maps	• Highlights regions with anticipated discrepancies due to voxel-wise uncertainties	+ Spatially resolved dosimetric uncertainty information
		• Voxel-wise uncertainties can be based on geometric factors (Salguero *et al* 2011), principal component analysis (Murphy *et al* 2012) or stochastic methods (Hub *et al* 2012)	+ No reference required
			− Previous DIR uncertainty measure required
	Energy-conservation-based criterion	• Structure-wise comparison of delivered energy with the energy of the warped representation of the dose (Zhong and Chetty 2017, Wu *et al* 2023)	+ Reliability measure for regions with mass/volume change
			− References required
			− Only structure-wise information

In this review, we refer to dose mapping as the process of warping/projecting/transferring a dose distribution, defined in one image, to a second image of the same patient. We refer to dose accumulation as the summation of the mapped dose distribution and a secondary dose distribution defined in the second image. Quantifying the correctness of dose mapping is challenging but essential in RT (Murr *et al* 2023). Some authors suggest using TG-132 thresholds (Xiao *et al* 2020), but the TG-132 report explicitly states ‘[t]he use of deformable registration for dose accumulation … is outside of the scope of this task group.’ (Brock *et al* 2017). For this reason, we feel that the metrics and thresholds proposed by TG-132 are not sufficient to evaluate image registration for dose mapping/accumulation. Instead, dosimetry uncertainty measures for clinical practice are needed.

#### 4.2.1. Correlation within measures

Geometric measures are not independent and self-correlate. Loi *et al* found a linear relationship between mean distance to agreement (MDA) and dice similarity coefficient (DSC) (Loi *et al* 2018). Also, a correlation between distance discordance metric (DDM) and Harmonic energy (HE) has been found (Kierkels *et al* 2018). Reporting multiple measures is still useful despite being redundant. For example, the DSC limitations can be critically analysed in conjunction with other metrics, such as MDA for different structures and volumes (Jena *et al* 2010, Brock *et al* 2017, Loi *et al* 2018). Combining different geometrical metrics can improve the understanding of the overall quality of the DIR for a specific application.

Different implementations and specific ways to use the same measure can lead to vastly different results. For example variations of up to 50% in DSC, 50% in Hausdorff distance (HD) and 200% in MDA were found between the same structure sets, evaluated by different institutions (Gooding *et al* 2022). Comparing results from different studies and centres should therefore be taken with care. The correlation between geometric and dosimetric measures was found to be low (Hvid *et al* 2016, Pukala *et al* 2016, Poel *et al* 2021, Nash *et al* 2022, Kamath *et al* 2023).

### 4.3. AI/DL-based uncertainty quantification

Further to its implementation as a DVF generator for the registration process, DL can also be used for the quantification or prediction of uncertainties in DIR (Smolders *et al* 2022b, 2022a, 2023a). DSM that are not used in the optimization of output DVFs, provide further uncertainty quantification metrics that can be used to determine the quality of the overall registration and highlight regions of poor accuracy (Galib *et al* 2020). The implementation of algorithms for automated image segmentation allows for the potential use of reference-based DIR evaluations (table 1) with limited or no user interaction. In this case the segmented structures must be consistent between the datasets used in the image registration. Additionally, DL-based DIR showed the potential of having inherent uncertainty assessments within the DL framework (Grigorescu *et al* 2021, Gong *et al* 2022, Khawaled and Freiman 2022b).

### 4.4. Treatment margins

Uncertainties in any DIR-facilitated process that is used to generate contours (e.g., image registration for standard treatment planning or atlas-based segmentation) should be quantified and included in the treatment margins. To achieve this, population-based studies would be required where the calculated uncertainties can be used in the margin formula (van Herk *et al* 2000). However, guidelines detailing the quantification and inclusion of these uncertainties are missing.

## 5. Application-specific DIR uncertainty

In this chapter, studies investigating the effect of DIR uncertainties for the deformation of images, structures and doses used in RT are reviewed (figure 1).

### 5.1. Deformed images

#### 5.1.1. Applications at planning

The TG-132 report and other recommendations suggest imaging the patient in the treatment position whenever possible to minimise the magnitude of the required deformation during registration (Brock *et al* 2017, Barber *et al* 2020).

#### 5.1.2. Intrafraction applications

DIR has been used to derive motion-corrected images from 4D CT scans (Wolthaus *et al* 2008) with average landmark-position differences of 0.5 mm for all directions in the tumour region. DIR is also used to reconstruct time-resolved 4D MRI (Nie *et al* 2020), with reported centre of mass differences of 2.9±0.6 mm. We expect the geometrical uncertainties of propagated images to be similar to those of structure propagation, considering both utilise the same input data.

#### 5.1.3. Interfraction applications

With MRI linac or CBCT-based online adaptation becoming more commonly available, the interest in deforming images between fractions for dose calculation and optimization is increasing (Kraus *et al* 2017, Tenhunen *et al* 2018, Irmak *et al* 2020, Byrne *et al* 2021). In these workflows, the calculated dose distribution is unlikely to be accurate considering the spatial uncertainties in the deformed CT, especially in areas with large density changes.

To correct for density changes that are not represented by the deformed image such as moving air in the gastro-intestinal organs, the density in these areas is often overwritten with the density of air or water (van Timmeren *et al* 2020). Research investigating the impacts of these overwrites on photon RT has found these impacts to be not clinically relevant (Pham *et al* 2022), except for very large air cavities (Thapa *et al* 2019). For protons, these density corrections are likely more relevant.

To avoid the use of DIR and manual density overwrites, direct dose calculation on the MRI or CBCT images has been investigated. The generation of synthetic CT images from MRI is reviewed elsewhere (Owrangi *et al* 2018, Hoffmann *et al* 2020, Boulanger *et al* 2021). Methods of scatter correction to make CBCT usable for dose calculation are widely explored (Kurz *et al* 2016, Giacometti *et al* 2019, Jarema and Aland 2019, Lalonde *et al* 2020, Trapp *et al* 2022).

#### 5.1.4. Intervention follow-up

Follow-up images after intervention can be registered to a planning CT to understand the relation and location of local failure such as recurrence or necrosis with a planned dose distribution and planning structures (Chang *et al* 2018, Kamal *et al* 2020, Abdel-Aty *et al* 2022). In these cases, dramatic changes are observed caused by the time between images, surgical intervention, or other medical issues. Systematic studies quantifying the impact of DIR uncertainties for intervention follow-up are rare and further work needs to be done to quantify and account for them in patterns of failure.

### 5.2. Propagated structures

#### 5.2.1. At planning

For treatment planning, structures are commonly defined on the planning CT. Structure definition can be challenging on CT due to low contrast compared to other imaging modalities such as MRI. Including multiple imaging modalities for contouring can lead to a reduction in inter-observer variability (Caldwell *et al* 2001, Farina *et al* 2017, Hall *et al* 2018). Though it is common to merge CT with PET, MRI or other images for contouring, the effect of deformable registration errors is not well investigated. (Barber *et al* 2020) therefore suggested using rigid registration wherever possible.

DIR is also used in atlas-based auto-segmentation, which is increasingly used in clinics to assist contouring. In this case, DIR is applied between images from different patients (Vrtovec *et al* 2020). Research showing a time benefit in using altas-based contours also show the necessity of manual corrections (Gooding *et al* 2013, Cardenas *et al* 2019, Welgemoed *et al* 2023). To our knowledge, there are no systematic studies on the impact of DIR implementation and DIR uncertainty for atlas-based segmentation. Studies do however investigate the impact of atlas selection (Schipaanboord *et al* 2019) or institution-specific implementation (Gooding *et al* 2013). As an alternative to atlas-based auto-segmentation, DL-based auto-segmentation was developed. Different auto-segmentation methods are reviewed elsewhere (Cardenas *et al* 2019, Schipaanboord *et al* 2019, Vrtovec *et al* 2020, Harrison *et al* 2022). The details of auto segmentation methods are out of the scope of this study.

#### 5.2.2. Intrafraction applications

DIR is used to map contours between different breathing phases, or intrafraction changes in patient treatment positions, to reduce the time needed for contouring. In clinical practice, the propagated structures are visually checked and corrected if necessary (Gaede *et al* 2011, Peroni *et al* 2013, Liu *et al* 2016, Ma *et al* 2017, Willigenburg *et al* 2022).

#### 5.2.3. Interfraction applications

Structure propagation can speed up recontouring for repeated imaging of a patient (Sonke *et al* 2019). This can be used for evaluation of recalculated doses or adaptive planning, but it is especially important for online adaptive workflows. The clinical availability of regular or daily imaging, such as scheduled repeated CT or daily CBCT, and the implementation of online adaptive workflows has led to multiple studies on the quality of deformed structures for adaptive planning.

Table 2 summarises recent studies investigating geometric DIR uncertainties for structure propagation for different anatomical sites and imaging modalities. The structures have been evaluated using geometrical measures introduced in table 1. Commercial and open access algorithms show similar performance (Scaggion *et al* 2020b). The majority of studies incorporate reference structures of a single expert physician. Variations between structures defined by different physicians are observed in many studies, and these inter-expert structure variations are currently de-facto the clinically accepted variability. Research comparing DIR propagated structure uncertainties to physician-to-physician uncertainties, has demonstrated results approaching inter-expert contour variation (Riegel *et al* 2016, Woerner *et al* 2017, Rigaud *et al* 2019, Nash *et al* 2022).

**Table 2.** Literature review of quantified geometric uncertainties in DIR-facilitated processes for different anatomical regions and imaging modalities.

Indication	Image modality	DIR algorithm and/or vendor	Assessment method	Study type	DICE	HD	others (TRE, MDA, COM, …)	Reference
Brain	MRI to MRI	Demons, HAMMER, and state-of-the-art registration methods with integrated learned features from unsupervised deep learning. ICA: Independent Component Analysis	Compare to segmented structures in the datasets	IXI dataset and ADNI dataset	XI dataset:			(Wu *et al* 2013)
					Demons: 0.752			
					M+PCA: 0.790			
					M+ISA: 0.789			
					HAMMER: 0.789			
					H+PCA: 0.754			
					H+ISA: 0.801			
					ADNI dataset:			
					Demons: 0.869			
					M+PCA: 0.789			
					M+ISA: 0.844			
					HAMMER: 0.821			
					H+PCA: 0.820			
					H+ISA: 0.873			
Brain	MRI to MRI	ANTs, VoxelMorph-1 (DL-based), VoxelMorph-2 (DL-based)	Compare to segmentations performed by FreeSurfer checked by visual inspection	7829 T1 weighted brain MRI scans from eight publicly available datasets	Average DICE: Affine only: 0.567			(Balakrishnan *et al* 2018)
					ANTs: 0.749			
					VoxelMorph-1: 0.724			
					VoxelMorph-2: 0.750			
Brain	MRI to MRI	Cue-Aware Deep Regression Network (DL-based)	Compare to segmented structures in the dataset	Three databases, i.e. LONI LPBA40, IXI, and ADNI	Average overall DICE: 0.7526		Average surface distance (ASD) in mm: Overall ≈ 0.6–0.7 (25th-75th percentile)	(Cao *et al* 2018)
Brain	MRI to MRI (2D)	Unsupervised DL-based (Bayesian Framework)	Compare to 4 largest anatomical structures in the reference dataset	MGH10 dataset, 10 subjects, 10 slices each	Overall DICE			(Khawaled and Freiman 2020)
					VoxelMorph: 0.7109			
					Proposed: 0.736			
Brain	Inter-patient MRI	TransMorph: Transformer for unsupervised image	Inter-patient MRI: compare to 30 anatomical structures labeled by FreeSurfer	Inter-patient MRI: 260 T1-weighted brain MRI images from John Hopkins University	Please consult (Chen *et al* 2022) for en extensive comparison of DICE values	Please consult (Chen *et al* 2022)for en extensive comparison of HD values	Please consult (Chen *et al* 2022)for en extensive comparison of SDlogJ and SSIM values	(Chen *et al* 2022)
	Atlas-to- patient MRI XCAT-to-CT	registration	Atlas-to-patient MRI: compare to 30 anatomical structures labeled by FreeSurfer	Atlas-to-patient MRI:				
				576 T1-weighted brain MRI images from the IXI database				
				XCAT-to-CT: XCAT phantom and 50 non-contrast chest-abdomen-pelvis CT scans				
Head and neck	CT to CT	MIM, Velocity, Raystation, Pinnacle, Eclipse	Compared to physician drawn reference	10 virtual head and neck phantoms (DIREP)			Mean TRE: 0.5 mm − 3 mm	(Pukala *et al* 2016)
							Maximum TRE: 22 mm	
Head and neck	CT to CT	MIM, Velocity, Eclipse	Compared to physician drawn reference	35 institutions, 10 virtual head and neck phantoms (DIREP)			Mean TRE:	(Kubli *et al* 2021)
							Velocity 2.04±0.35 mm;	
							MIM 1.10±0.29 mm; Eclipse 2.35±0.15 mm	
							All mean TRE < 3 mm	
							Maximum errors > 2 cm	
Head and neck	CT to CT	Raystation (simple Anaconda, detailed Anaconda, simple Morfeus, detailed Morfeus)	Compared to physician drawn reference	10 head and neck cancer patients	GTV DSC: Simple Anaconda 0.78 ± 0.11; Detailed Anaconda 0.96 ± 0.02; Simple Morfeus 0.64 ± 0.15; Detailed Morfeus 0.91 ± 0.03; Larger DSC for OARs larger than the eye compared to smaller OARs			(Zhang *et al* 2018)
Head and neck	CT to CT	10 DIR combinations using demons and free form deformations (FFD)	Compared against each other and 2 expers using landmarks	15 patients, 6 weekly CTs			Landmark Registration Error: interobserver distance 2.01 mm (1.29 mm), most effective DIRs 2.44 mm (and 1.30 mm)	(Rigaud *et al* 2019)
Head and neck	CT to CBCT	NiftyReg	Compared to physician drawn reference	5 head and neck patients	Mean DSC: 0.850 External contour: 0.986			(Veiga *et al* 2014)
					Bony anatomy: 0.846			
					Soft tissue: 0.790			
					(DIR better than rigid registration)			
Head and neck	CT to CBCT	Five commercially available DIRs (RayStation, ADMIRE, Mirada, ProSoma, Pinnacle)	Compared to physician drawn and STAPLE reference	10 head and neck patients: 5 oropharyngeal, 2 oral cavity, 1 hypopharynx, 1 supraglottic and 1 of unknown primary (target below nasal region)	clinician drawn reference: Brainstem 0.68(0.09), Spinal Cord, 0.62(0.14), Larynx 0.75(0.1), Left Parotid 0.72 (0.08), Right Parotid 0.76(0.06) STAPLE reference: Brainstem 0.93(0.04), Spinal Cord 0.87(0.04), Larynx 0.93(0.04), Left Parotid 0.93 (0.06), Right Parotid 0.92(0.03)	clinician drawn reference: Brainstem 10.8(3.5), Spinal Cord,7.1(2.8), Larynx 10.2(4.5), Left Parotid 12.9(4.8), Right Parotid 12.2(3.9) STAPLE reference: Brainstem 4.4(2.7), Spinal Cord 4.3(2.7), Larynx 3.5(1.1), Left Parotid 3.5(1.1), Right Parotid 3.4(1.1)	MDA: clinician drawn reference: Brainstem 2.9(0.1), Spinal Cord, 1.5(0.5), Larynx 2.2(1.1), Left Parotid 2.2(0.5), Right Parotid 2.0(0.5) STAPLE reference: Brainstem 0.8(0.5), Spinal Cord, 0.5(0.2), Larynx 0.5(0.3), Left Parotid 0.5(0.2), Right Parotid 0.5(0.2) Centroid separation in mm: clinician drawn reference: Brainstem 5.7(2.9), Spinal Cord, 9.7(5.8), Larynx 3.2(2.7), Left Parotid 3.6(1.6), Right Parotid 3.1(1.4) STAPLE reference: Brainstem 1.6(1.3), Spinal Cord, 2.8(2.1), Larynx 0.9(1.0), Left Parotid 0.9(0.6), Right Parotid 0.9(0.6)	(Nash *et al* 2022)
Head and neck	CT to CBCT	MIM DIR	Compared to physician structures	30 HN patients, squamous cell carcinoma of the oral cavity, pharynx or larynx, DIR to first and last CBCT	First CBCT Parotid L 0.95 Parotid R 0.95 Submandibular L 0.91 Submandibular R 0.93 Esophagus 0.85 Spinal cord 0.89 Last CBCT Parotid L 0.95 Parotid R 0.95 Submandibular L 0.85 Submandibular R 0.87 Esophagus 0.84 Spinal cord 0.87	First CBCT Parotid L 0.7 cm Parotid R 0.7 cm Submandibular L 0.6 cm Submandibular R 0.6 cm Esophagus 0.5 cm Spinal cord 0.3 cm Last CBCT Parotid L 0.7 cm Parotid 0.7 cm Submandibular 0.7 cm Submandibular 0.7 cm Esophagus 0.8 cm Spinal cord 0.3 cm		(Hvid *et al* 2016)
Head and neck	CT to CBCT	10 DIRs (optical flow, Demons, Level set, Spline)	Compared to physician reference	21 HN patients	data not shown in tables, please refer to the plots in the paper.	data not shown in tables, please refer to the plots in the paper.		(Li *et al* 2017)
Head and neck	MRI to MRI	Monaco DIR	Compared to manual defined structures and intra observer variability	17 patients, larynx (3), oropharynx (10), oral cavity (1) and hypopharynx (3), planning MRO + 3 repeated MRI	MRI to MRI GTV-T 0.55 GTV-N 0.58 Brain Stem 0.89 Spinal cord 0.86 R parotid 0.81 L parotid 0.82 R submand 0.77 L submand 0.78 Thyroid 0.74 IOV GTV-T 0.68 GTV-N 0.72 Brain Stem 0.96 Spinal cord 0.89 R parotid 0.93 L parotid 0.88 R submand 0.89 L submand 0.88 Thyroid 0.81	IOV GTV-T 9.8 mm GTV-N 5.0 mm Brain Stem 3.0 mm Spinal cord 2.8 mm R parotid 3.7 mm L parotid 4.4 mm R submand 3.1 mm L submand 3.3 mm Thyroid 4.3 mm MRI to MRI GTV-T 7.6 mm GTV-N 5.7 mm Brain Stem 4.3 mm Spinal cord 5.0 mm R parotid 7.7 mm L parotid 7.1 mm R submand 5.0 mm L submand 4.6 mm Thyroid 7.2 mm	mean surface distance, IOV GTV-T 2.2 mm GTV-N 1.1 mm Brain Stem 0.2 mm Spinal cord 0.5 mm R parotid 0.4 mm L parotid 0.8 mm R submand 0.5 mm L submand 0.6 mm Thyroid 0.8 mm MRI to MRI GTV-T 2.0 mm GTV-N 1.6 mm Brain Stem 1.0 mm Spinal cord 0.6 mm R parotid 1.2 mm L parotid 1.1 mm R submand 1.1 mm L submand 0.9 mm Thyroid 1.4 mm	(Christiansen *et al* 2021)
Head and neck, thorax, pelvis	CT to CT	Velocity	Compared to two observers	30 head and neck and 20 prostate cancer patients		mean HD, structure dependence HN Intraobserver variation 0.7mm-2.3 mm, interobserver variation 1.0mm-5.0 mm, DIR error 1.1mm-3.0 mm Pelvis Intraobserver variation 1.3mm-2.5 mm, interobserver variation 1.6mm-3.1 mm, DIR error 1.9mm-3.1 mm		(Riegel *et al* 2016)
Head and neck, thorax, pelvis	CT to CT	RayStation, MIM, Velocity AI and Smart Adapt, Mirada XD, ABAS	Compared to reference contours generated with a ground truth DVF	synthetic CT images (simQA), thirteen institutions	HN 0.84–0.93 Thorax 0.52–0.97 Pelvis 0.45–0.87		Mean Distance to Conformity (MDC) in mm HN 2.26–3.36 Thorax 2.38–4.57 Pelvis 3.69–6.03	(Loi *et al* 2018)
Head and neck, pelvis	CT to CT	MIM-Maestro, Raystation, Velocity	Compared to reference contours generated with a ground truth DVF	9 pairs of synthetic CTs (simQA)	trachea, esophagus, spinal cord, and spinal canal 0.95–0.98 pituitary 0.34–0.92		MDA (mm): trachea, esophagus, spinal cord, and spinal canal 2.10–2.70 pituitary 3.02–3.81	(Shi *et al* 2021)
Head and neck, Prostate, Pancreas	CBCT to CT	Physician-to-physician, Velocity	Compared to physician drawn reference	HN 6 patients, prostate 5 patients, pancreas 5 patients	HN Mean DSC: Physician-to-physician 0.87 DIR 0.77 Prostate Mean DSC: Physician-to-physician 0.9 DIR 0.74 Pancreas: Mean DSC: Physician-to-physician 0.93 DIR 0.84	All: Mean HD: Physician-to-physician 11.32 mm Rigid 12.1 mm DIR 12.0 mm		(Woerner *et al* 2017)
virtual phantoms and brain, HN, cervix, prostate	CT to CT	Smart Adapt (Eclipse)	Compared to physician structures	10 virtual phantoms, and brain (n = 5), HN (n = 9), cervix (n = 18) and prostate (n = 23) patients	Brain 0.91 (0.04) HN 0.84 (0.03) Prostate 0.81 (0.05) Cervix 0.77 (0.05) per-structure DSCs in paper	Brain 1.37 (0.97) HN 1.06 (0.22) Prostate 2.70 (0.24) Cervix 3.23 (0.78) per-structure HD in paper	Center of mass, Brain 1.69 (0.84) HN 1.63 (0.30) Prostate 5.19 (1.34) Cervix 5.79 (1.42) per-structure COM in paper	(Jamema *et al* 2018)
Abdominal, Head and neck, Thoracic	4DCT	Mirada	Compared to physician drawn reference	3 abdominal patients, 7 thoracic patients, two images from extreme respiratory phases	Abdominal: Nearly all OARs DSC > 0.90, pancreas 0.74-0.88 HN: Lower DSC, lowest for pharyngeal constrictor low contrast in this region, small size of structure and proximity to air cavities, Thorax: Nearly all OARs DSC > 0.90, esophagus 0.79-0.85		Thoracic: Mean TRE: 3.4–8.9 mm (above AAPM report recommendation) Maximum TRE: 10.1–29.0 mm	(Latifi *et al* 2018)
Retina and Heart	Retina: Colour fundus images to fluorescein angiography Heart: MRI to MRI	GAN (DL-based)	Retina: Compare to registration ground-truth derived with ITK Heart: Compare to manual segmented structures	Retina: 26 image pairs Heart: Sunybrook cardiac dataset, 45 cardiac cine MRI scans (short-axis cardiac image slices each containing 20 timepoints)	Average DICE: Retina GAN: 0.946 DIRNet: 0.911 Elastix: 0.874 Before registration: 0.843 Heart: GAN: 0.85 DIRNet: 0.80 Elastix: 0.77 Before registration: 0.62	HD95 (95th percentile HD): Retina GAN: 4.2 DIRNet: 5.9 Elastix: 9.7 Before registration: 11.4 Heart: GAN: 3.9 DIRNet: 5.03 Elastix: 5.21 Before registration: 7.79	Mean absolute surface distance (MAD): Retina GAN: 3.1 DIRNet: 5.0 Elastix: 8.7 Before registration: 9.1 Heart: GAN: 1.3 DIRNet: 1.83 Elastix: 2.12 Before registratio	(Mahapatra *et al* 2018)
abdomen, thorax, pelvis	4DCT, MR-MR, CT-MR	Morpheus (Raystation)	Compared to manually defined contours and langmarks	74 patients, thoracic and abdominal 4DCT and MR,, liver CT-MR, prostate MR-Mr			mean DTA <1 mm for controlling strucutres and 1.0–3.5 mm for implicitly deformed strucutres TRE: 2.0 mm − 5.1 mm	(Velec *et al* 2017)
Thorax/Esophagus	4DCT	Bspline (Velocity), free form deform (FDD), Horn-Schunk optical flow (OF), Demons	Compared to manual landmarks	5 esophagus patients from DIR lab dataset			3D registration errors B-spline 1.84 (0.97)−3.72 (3.17) mm FDD 2.49 (1.21)−4.52 (3.45) OF 1.42 (0.92)−3.40 (2.93) Demons 1.40 (0.96)−4.39 (4.23)	(Kadoya *et al* 2014)
Thorax	CT to CT	4 RayStation (RaySearch 5 MIM Software (Cleveland, OH), 3 used Velocity	Compared to expert defined anatomical landmarks (DIR-Lab references)	10 patients with esophageal or lung cancer			3D registration error RayStation 1.26–3.91 mm, MIM 2.17–3.61 mm Velocity 4.02–6.20 mm	(Kadoya *et al* 2016)
Lung	CT to CT	Demons, Salient Feature BAsed registration (PInnacle), Morphons	Compared to physician structures	17 NSCLC patients, 4D CTs (50% exhale was used)	data not shown in tables, please refer to the plots in the paper.	data not shown in tables, please refer to the plots in the paper.	COM GTV-tumor 0.27–0.29 cm nodal-GTVs 0.31–0.37 cm	(Hardcastle *et al* 2013)
Lung	4DCT-4DCBCT	Demons, SICLE	Compared to physician drawn reference	10 locally advanced non-small cell lung cancer patients, one 4D fan-beam CT and 7 weekly cone-beam CT; Day-to-day and phase-to-phase registrations	Day-to-day Mean DSC: SICLE 0.75 Demons 0.70 Rigid-tumor registration 0.66 Rigid-bone registration 0.6 Phase-to-phase (4D CBCT): SICLE 0.8 Demons: 0.79			(Balik *et al* 2013)
Lung	4DCT	In-house Bspline, MIM freeform	Compared to physician drawn reference	4D-CTs of 12 lung cancer patients acquired in prone and supine positions	Mean DSC: In-house Bspline 0.8 MIM 0.8	Mean HD: In-house Bspline 22.5 mm MIM 22.6 mm	Mean MDA: In-house Bspline 2.3 mm MIM 2.1 mm	(Guy *et al* 2019)
Lung	4DCT	10 DIR algorithms (optical flow, demons)	Compared to physician defined reference/fiducials (FM)	5 patients implanted fiducial markers (FM) as ground truth			TRE FM positions 1.82–1.98 mm tumor position TREs 1.29–1.78 mm	(Han *et al* 2022)
Heart	MRI to MR	SVF-Net (DL-based)	Compare to segmented structures in the dataset	187 3D MRI cardiac images	No numbers reported, only plots, box-plot (25%-75%): Left ventricle myocardium ≈ 0.75–0.8 Right ventricle myocardium ≈ 0.45–0.55 Left ventricle blood pool ≈ 0.85–0.9 Right ventricle myocardium ≈ 0.75–0.85	No numbers reported, only plots, box-plot (25%-75%): Left ventricle myocardium ≈ 4–5.5 mm Right ventricle myocardium ≈ 5–6 mm Left ventricle blood pool ≈ 4–5 mm Right ventricle myocardium ≈ 4.5–6 mm		(Rohé *et al* 2017)
Cervical cancer	CT to CT	Velocity, Elastix	Compared to physician drawn reference	5 cervical brachytherapy patients	Mean DSC: Bladder Velocity 0.85 Rectum Velocity 0.72 Rectosigmoid Velocity 0.47 Bladder Elastix 0.76 Rectum Elastix 0.68 Rectosigmoid Elastix 0.50	Mean HD Rectosigmoid Velocity 35.94 mm Rectosigmoid Elastix 40.76 mm		(Belon *et al* 2015)
Intraheptic cholangiocarcinoma (IHCC)	CT to CT	Five commercially available DIRs (Demons, B-splines, salient feature-based, anatomically constrained, finite element-based algorithm)	Compared to physician drawn reference	29 IHCC patients			Mean TRE: Demons 4.6±2.0 mm; B-splines 7.4±2.7 mm; salient feature-based 7.2±2.6 mm; anatomically constrained 6.3±2.3 mm; finite element-based 7.5±4.0 mm; Maximum errors > 1 cm for all techniques	(Sen *et al* 2020)
Liver	CT to CT	MIM , Velocity.,	Compared to fiducial markers (FM) as ground truth	24 Patients with liver tumor, pre and post treatment images (median 10 months)			FM error MIM: 0.4–32.9 (9.3 ± 9.9) mm Velocity 0.5–38.6 (11.0 ± 10.0) mm	(Fukumitsu *et al* 2017)
Liver	CT to CT	Unsupervised Cycle-Consistent CNN (DL-based)	Compare to 20 anatomical points in the liver and adjacent organs marked by radiologists	Liver cancer (HCC) patients at Asan Medical Center, Seoul, South Korea: 555 scans for training, 50 scans for testing			TRE Arterial to Portal Elastix: 3.26 VoxelMorph: 6.67 CNN: 4.91 Delayed to Portal Elastix: 2.96 VoxelMorph: 5.35 CNN: 3.76% of Jacobian determinant ≤ 0 Arterial to Portal VoxelMorph: 0.0327 CNN: 0.0175 Delayed to Portal VoxelMorph: 0.0311 CNN: 0.0181 NMSE (normalized mean square error) Arterial to Portal VoxelMorph: 0.0278 CNN: 0.0277 Delayed to Portal VoxelMorph: 0.0213 CNN: 0.0199	(Kim *et al* 2019)
pancreatic	CT to CBCT	B-spline registration mutual-information (MI), mattes mutual-information (mattes) and gradient magnitude (GM) and also different regularization levels *λ* ∈ {0.05; 0.005; 0.00025}, GM(*λ* = 0.05),	Compared to physician drawn reference	Fifteen pancreatic cancer patients			best registration outcome for the visual comparison, the lowest median deviation was obtained with GM(*λ* = 0.005) and GM(*λ* = 0.05), whereas the variation over the patient collective was much smaller for GM(*λ* = 0.05).	(Ziegler *et al* 2019)
Prostate	CT to ultrasound	Rigid, MIM		10 prostate patients, HDR-brachy therapy	Mean DSC: Rigid 0.78 ±0 .06 DIR 0.93 ± 0.01	Mean HD: Rigid 11.64 ± 2.38 mm DIR 5.19 ± 1.47 mm	Mean MDA: Rigid 2.50 ±0 .70 mm DIR 0.69 ± 0.06 mm	(Vozzo *et al* 2021)
Prostate	CT to CT	intensity based Elastix	Compared to manual delineation	18 prostate cancer patients, 7–10 repeat CT	prostate 0.87 ± 0.05, seminal vesicles 0.63 ± 0.18, lymph nodes 0.89 ± 0.03, Rectum 0.76 ± 0.06, Bladder 0.86 ± 0.09	95 percentile HD prostate 3.35 ± 1.19 mm, seminal vesicles 4.76 ± 2.77 mm, lymph nodes 3.57 ± 0.99 mm, Rectum 10.83 ± 5.93 mm , Bladder 8.91 ± 6.76 mm	mean surface distance (MSD) prostate 1.42 ± 0.48 mm, seminal vesicles 1.97 ± 1.22 mm, lymph nodes 1.46 ± 0.44 mm, Rectum 3.29 ± 1.31 mm , Bladder 2.92 ± 1.90 mm	(Qiao *et al* 2019)
Prostate	CT to CT	improved AI DIR in Elastix	Compared to manual delineation	evaluation on 2 datasets 14+18 patients, follow up on Quiao *et al*, improved adaptive dose constraints with this one	results on two datasets Prostate 0.87 ± 0.08/0.87 ± 0.12 seminal vesicles 0.70 ± 0.13/0.75 ± 0.18 Lymph nodes 0.87 ± 0.07/ – Rectum 0.82 ± 0.12/0.78 ± 0.15 Bladder0.89 ± 0.12/0.83 ± 0.17	results on two datasets in mm Prostate 3.07 ± 1.30/3.93 ± 2.24 seminal vesicles 3.82 ± 3.19/4.92 ± 5.13 Lymph nodes 3.74 ± 1.02/ – Rectum 8.66 ± 6.92/10.4 ± 7.77 Bladder 5.11 ± 4.38/11.5 ± 12.5	mean surface distance (MSD) results on two datasets in mm Prostate 1.29 ± 0.39/1.54 ± 0.67 seminal vesicles 1.48 ± 1.16/1.67 ± 1.38 Lymph nodes 1.49 ± 0.44/ – Rectum 2.39 ± 1.92/2.67 ± 1.76 Bladder 1.72 ± 1.17/3.89 ± 4.00	(Elmahdy *et al* 2019)
prostate	CT-CBCT	anaconda	Compared to physician drawn reference	10 prostate patients	body ROI controlling: prostate 0.84 ± 0.05 rectum 0.75 ± 0.05 bladder 0.69 ± 0.07 seminal vesicles 0.65 ± 0.11 all ROIs controlling: prostate 0.98 ± 0.00 rectum 0.97 ± 0.01 bladder 0.98 ± 0.00 seminal vesicles 0.94 ± 0.03		COM body ROI controlling (mm): prostate 2.0 ± 1.5 rectum 3.7 ± 1.4 bladder7.8 ± 2.2 seminal vesicles 3.6 ± 1.2 all ROIs controlling (mm): prostate 0.1 ± 0.0 rectum 0.3 ± 0.2 bladder 0.2 ± 0.1 seminal vesicles 0.6 ± 0.6	(Takayama *et al* 2017)
Prostate	CT to CT and CT to CBCT	3 DIR algorithms implemented in MIM (DIR Profile, normalized intensity-based (NIB) and shadowed NIB DIR algorithms)	Compared to manually drawn reference	20 patients (453 fractions)	CT to CT bladder: 0.729–0.943 rectum: 0.737–0.913 CT to CBCT bladder: 0.713–0.906 rectum: 0.710–0.879	CT to CT bladder: 7.26–18.40 mm rectum: 9.63–16.37 mm CT to CBCT bladder:12.24–22.57 mm rectum: 11.25–18.49 mm	MDA in mm CT to CT bladder:0.86–4.47 rectum: 0.89–2.96 CT to CBCT bladder:1.51–4.68 rectum: 1.31–3.29	(Hammers *et al* 2020)
Prostate	CT to MRI, MRI to MRI	Monaco DIR	Compared to manual defined structures and intra observer variability	12 high-risk prostate cancer patients, prostate and pelvic lymph nodes treated on MRI linac	CT to MRI Prostate 0.84, Seminal Vesicles 0.68, Rectum 0.77, Bladder 0.87, R fem. Head 0.93, L fem. Head 0.91 MRI to MRI Prostate 0.90, Seminal Vesicles 0.76, Rectum 0.87, Bladder 0.92, R fem. Head 0.95, L fem. Head 0.94 Inter observer Prostate 0.92, Seminal Vesicles 0.81, Rectum 0.95, Bladder 0.97, R fem. Head 0.95, L fem. Head 0.94	CT to MRI Prostate 7.16 mm, Seminal Vesicles 6.55 mm, Rectum 12.36 mm, Bladder 10.88 mm, R fem. Head 4.96 mm, L fem. Head 4.98 mm MRI to MRI Prostate 5.10 mm, Seminal Vesicles 5.54 mm, Rectum 8.89 mm, Bladder 5.71 mm, R fem. Head 4.77 mm, L fem. Head 4.75 mm Inter observer Prostate 4.89 mm, Seminal Vesicles 5.31 mm, Rectum 07.65 mm, Bladder 4.05 mm, R fem. Head 4.41 mm, L fem. Head 5.21	mean surface distance, CT to MRI Prostate 1.6 mm, Seminal Vesicles 1.48 mm, Rectum 2.41 mm, Bladder 1.96 mm, R fem. Head 1.09 mm, L fem. Head 1.37 mm MRI to MRI Prostate 1.00 mm, Seminal Vesicles 1.17 mm, Rectum 1.25 mm, Bladder 1.11 mm, R fem. Head 0.81 mm, L fem. Head 0.81 mm Inter observer Prostate 0.88 mm, Seminal Vesicles 0.86 mm, Rectum 0.65 mm, Bladder 0.55 mm, R fem. Head 0.75 mm, L fem. Head 1.05 mm	(Christiansen *et al* 2020)
Prostate	MRI to transrectal ultrasound	Weakly-supervised CNN (DL-based)	Compare to manually segmented structures	108 pairs of T2-weighted MR and TRUS images	Composite-Net Median: 0.82 Percentiles [25th, 75th]: [0.78,0.86]		TRE (mm): Composite-Net Median: 4.7 Percentiles [25th, 75th]: [3.3,7.5]	(Hu *et al* 2018, p 218)
Phantoms		Elastix, BRAINS, Plastimatch, Raystation	Compared to results from synthetic image datasets from applying synthetic DVFs	4 computational anthropomorphic phantoms	Mostly DSC > 0.85 Only smallest structures mild failure DSC < 0.75		In case of severe deformations MDC > 3 mm	(Scaggion *et al* 2020a)

DIR: deformable image registration, HN: Hausdorff distance, TRE: target registration error, MDA: mean distance to agreement, COM: center of mass, HD: Hausdorff distance

Currently, there is no consensus on the use of DIR propagated structures for plan adaptation in the literature. Some authors conclude that propagated structures can be used for reoptimization and/or dose evaluation (Beasley *et al* 2016, Hvid *et al* 2016, Qiao *et al* 2019, Nenoff *et al* 2021b, Nash *et al* 2022), while others found that manual corrections are still necessary (Li *et al* 2017, Christiansen *et al* 2020, 2021). Generally, the literature agrees that a visual inspection of the DIR propagated structures remains necessary for dose evaluation and optimization. Furthermore, it has been observed that for most organs at risk (OARs) geometric uncertainties correlated only weakly to dosimetric errors (Hvid *et al* 2016, Pukala *et al* 2016, Nash *et al* 2022).

There are a small number of studies evaluating the dosimetric effect of uncertainties in propagated structures for dosimetric evaluation or plan optimization during adaptive RT (table 3). For pancreas stereotactic body radiotherapy (SBRT), physician-drawn structures were compared to propagated structures by MIM and Precision DIR algorithms (Magallon-Baro *et al* 2022). They compared uncorrected propagated structures with physician-drawn structures in 0.5, 1 and 3 cm distance rings from the target. They found that replanning with uncorrected propagated structures improves the target coverage and OAR sparing compared to no adaptation. For the majority of fractions, manual correction of propagated structures could be avoided or be limited to the region closest to the target. Ray at al. evaluated the use of automatic deformed CTVs compared to physician defined CTVs and proposed a framework to determine PTV margins based on automatic deformed CTVs for adaptive planning (Ray *et al* 2020). Nash *et al* showed that even large geometrical structure differences rarely had a statistically significant impact on OAR dose-volume-histograms (DVH) parameters and concluded that DIR propagated structures are suitable for dose evaluation (Nash *et al* 2022). Similar conclusions were found by Hvid *et al* (2016).

**Table 3.** Literature review of quantified dosimetric uncertainties in DIR-facilitated processes for different anatomical regions and imaging modalities. DIR: deformable image registration, SBRT: stereotactic body radiotherapy, VMAT: volumetric modulated arc radiotherapy, IMPT: intensity modulated proton therapy, DDM distance discordance metric.

	Application	Indication	Image modality	DIR algorithm and/or vendor	Assessment method	Study type	Dosimetric uncertainty					Reference
Structure propagation	Photons interfraction dose recalculation	Head and neck	CT to CBCT	MIM	compared to physician reference	30 head and neck patients, squamous cell carcinoma of the oral cavity, pharynx or larynx, DIR to first and last CBCT	Dose difference when dose is evaluated on propagated versus reference structures					(Hvid *et al* 2016)
							First CBCT	Last CBCT				
							Parotid L 0.1 Gy	Parotid L 0.1 Gy				
							Parotid R −0.1 Gy	Parotid R −0.1 Gy				
							Submandibular L 0.1 Gy	Submandibular L −0.3 Gy				
							Submandibular R 0.1 Gy	Submandibular R −0.5 Gy				
							Esophagus 0.0 Gy	Esophagus 0.3 Gy				
							Spinal cord 0.1 Gy	Spinal cord 0.0 Gy				
	Photons interfraction dose recalculation	Head and neck	CT to CBCT	Five commercially available DIRs (RayStation, ADMIRE, Mirada, ProSoma, Pinnacle)	compared to physician drawn and STAPLE reference	10 head and neck patients: 5 oropharyngeal, 2 oral cavity, 1 hypopharynx, 1 supraglottic and 1 of unknown primary (target below nasal region)	Spinal cord D1cc occasionally exceeds planning tolerance (44 Gy) by 7–250 cGy Brainstem D1cc occasionally exceeds planning tolerance (54 Gy) by (29–199 cGy) Despite poor geometric agreement, the DVH parameters of propagated contours gave a reliable estimate of the organ dose					(Nash *et al* 2022)
	Photon adaptive planning (cyberknife)	Pancreas	CT to CT	Precision, MIM	compared to physician reference	35 pancreas patients, 98 fx CTs, breathhold	Plans optimized on propagated and reference contours, evaluated on reference contours					(Magallon-Baro *et al* 2022)
							Dose difference between no adaptation and					
							a) Physician reference	b) Precision	c) MIM			
							PTV −2.0%	PTV −2.7%	PTV −5.1%			
							GTV −0.1%	GTV −0.4%	GTV −1.6%			
							Stomach V35 Gy −0.2 cc	Stomach V35 Gy −0.1 cc	Stomach V35 Gy −0.1 cc			
							Duodenum V35 Gy −0.4 cc	Duodenum V35 Gy −0.2 cc	Duodenum V35 Gy −0.2 cc			
	Proton adaptive planning	Prostate	CT to CT	Elastix	compared to manual delineation	18 prostate cancer patients, 7–10 repeat CT	Plans optimized on propagated and reference contours, evaluated on reference contours Propagated contours could be directly used for reoptimization (V95% ≥ 98% for each target volume) in 89% of cases					(Qiao *et al* 2019)
	Proton adaptive planning	Lung	CT to CT	Plastimatch (B-splines, demons, Velocity, Mirada, Raystation (Anaconda, Morfeus)	compared to physician reference	5 NSCLC patients with 9 repeated DIBH CTs	Plans optimized on propagated and reference contours, evaluated on reference contours 0.04% average difference in CTV V95 with DIR versus 0.06% with rigid propagation and 9.7% without adaptation					(Nenoff *et al* 2021b)
	Proton adaptive planning	Lung & Head and neck	CT to CT	Rigid registration, Plastimatch B-splines, Commercial CNN, patient-specific CNN	autocontouring techniques compared to manual delineation	5 NCSLC patients 9 repeated CTs and 5 head and neck cancer patients with 4–7 repeated CTs	Plans optimized on automatic OARs contours showed small dependence on the contouring method (<5%). For automatic target contours the dosimetric effect can be larger than 5%. Compared to non-adaptive approaches the automatic contouring showed improved target coverage.					(Smolders *et al* 2023a)
Dose accumulation	Photon adaptive planning	Head and neck	CT to CT	Raystation Anaconda (simple & detailed) Raystation Morfeus (simple & detailed)	Not applicable	10 head and neck patients with weekly offline replanning	Deformed weekly doses accumulated and compared to primary planning dose Difference to primary planned dose:					(Zhang *et al* 2018)
								Simple Anaconda	Detailed Anaconda	Simple Morfeus	Detailed Morfeus	
							Homogeneity index	0.137 ± 0.115	0.006 ± 0.032	0.197 ± 0.096	0.006 ± 0.033	
							Main difference between simple and detailed algorithms.					
							Simple presetting: 344.6 cGy, 109.9 cGy, 329.0 cGy for D95, Dmean, Dmin in average					
							Detailed presetting: less than 20 cGy					
	Photon 4D dose calculation	Lung, liver	4DCT	6 open sourse algorithms from EMPIRE challenge (ANTS, VarReg, DIRART, NiftyReg, Elastix, Plastimatch)	Not applicable	5 patients with multiple lung metastasis, 5 patients with multiple liver metasatsis, VMAT	GTV D95% difference between plan on average CT and 4D dose simulation Lung metastasis: Variations mostly negligible (<0.5 Gy), but up to 7.85 Gy Liver metastasis: Lager variations more diverging, higher negative, up to −29.09 Gy					(Mogadas *et al* 2018)
	Photon 4D dose calculation	Lung	4DCT	SmartAdapt, Velocity, Anaconda (Raystation)	Not applicable	6 lung SBRT patients, VMAT	If results are limited to visually acceptable deformed images: Maximum difference in the evaluated DVH parameters was ≤3.0% for GTV D98, spinal cord D2%, heart D2% and ≤3.6% of the total structure volume for the ipsilateral lung					(Sarudis *et al* 2019)
	Proton 4D dose calculation	Liver	4DCT (generated from 4D MRI)	Plastimatch (B-splines, demons, in-house DIR, Mirada, Raystation (Anaconda, Morfeus)	Not applicable	9 liver cancer patients with generated 4DCTs, applying motion from 4DMRI, IMPT	CTV V95 differences up 11.34±12.57% for single fields without rescanning, large motion CTV V95 differences up to 3.46±1.40% for three-field plans with rescanning, large motion CTV V95 differences up to 0.37±0.38% for three-field plans with rescanning, small motion					(Ribeiro *et al* 2018)
	Photon dose calculation inter and intra-fraction	Lung	CT to CBCT	Admire (Eleta)	Not applicable	20 lung SBRT patients, comparison if inter- and intrafractional differences	95-percenteile of DDM (in mm) and dosimetric errors (in Gy)					(Huesa-Berral *et al* 2022)
							Structure	DDM Intrafraction in mm	DDM Interfraction in mm	DDM Interfraction dosimetric in Gy		
							GTV	0.93	1.54	1.67		
							Lung	1.86	2.16	0.86		
							Ribs	1.66	5.13	1.05		
							Heart	6.26	2.34	0.57		
							Esophagus	1.38	2.55	0.29		
							Spinal cord	0.16	8.00	1.28		
							The dosimetric impact of Interfraction changes is larger than intrafraction motion					
	Photon dose calculation	Abdomen	CT to CT	Thin Plate Spline—Robust Point Matching algortuhm with variable settings	Not applicable	16 liver SBRT patients, DIR uncertainty modeled by systematic variation of registration parameters	After selection of ‘realistic’ deformations, average difference between the 1st and 99th percentile of the cumulative maximum doses: 1.4 Gy for esophagus 0.7 Gy for stomach 0.9 Gy for duodenum (maximum difference for one patient: 3.3 Gy)					(Wang *et al* 2018)
	Tomotherapy	Head and neck	CT to megavoltage CT	PreciseART (Accuray)	Not applicable	20 Head and neck patients with daily MVCTs	Doses from daily MVCTs reconstructed and accumulated on the planning CT and compared to planned dose with warped contours on the daily MVCTs. Average dose uncertainty bounds (and confidence interval) for the cumulative treatment were: Parotids mean dose: 3.5% (97.1%–107.0%) Parotids D50%: 6.6% (98.2%–110.4%) Parotids V20Gy: 4.6% (95.6%–111.1%) PTV D95%: 0.4% (98.2%–100.2%)					(García-Alvarez *et al* 2022)
	Photon adaptive planning	Head and neck	CT to CBCT	4 different NiftyReg approaches	Not applicable	5 Head and neck cancer patients with weekly CBCTs	The four DIR methods resulted in similar geometrical matching, but smoothness and inverse consistency differed. The root mean squared dose difference of the different warped doses was 1.9%±0.8%. 9%±4% of voxels within the treated volume failed a 2% dose difference test, this value was larger in high dose gradient regions (21%±6%) and for poor CBCT quality regions (28%±9%).					(Veiga *et al* 2015)
	Photon VMAT	Head and neck	CT to CBCT	Bspline DIR , Varian’s demons DIR	Not applicable	12 Head and neck patients with 4 CBCTs	In-silico reference created with a B-spline algorithm. Inverse consisteny was assessed by forward and backward deformation. Dose was reconstructed by the demons algorithm and compared to the in-silico ground truth. 98.5% of all voxels were inverse consistent with the following confidence interval for the dose reconstruction of a single fraction relative to planned dose: Target structures: [2.3%; +2.1%] Critical OARs: [10.2%; +15.2%] Non-critical OARs: [9.5%; +12.5%] Inverse inconsistent voxels were associated with higher uncertainties.					(Lowther 2020a, 2020b)
	Photon dose calculation	Prostate	CT to CT	Demons algorithm	Not applicable	1 prostate patient with 9 CTs	Quantification of errors with unbalanced energy (UE) and compared to standard displacement error (SDE). High Pearson correlation above 70% between UE and SDE. Mean dose reconstruction error in target over nine fractions 1.68%.					(Zhong *et al* 2008)
	Photon dose calculation inter-fraction	Prostate	CT to CBCT	Demons algorithm	Not applicable	24 prostate patients with 8 weeklc CBCTs for 21 patients and daily CBCTs for 3 patients	Quantification of differences between planned and cumulated doses using DIR-based dose accumulation and quantifying the dose accumulation uncertainties with a numerical pelvis phantom. Standard deviation of the dose difference between planned and accumulated dose Mean bladder dose: 6.9 Gy Mean rectum wall dose: 2.0 Gy Dose accumulation uncertainty: Mean bladder dose: 2.7 Gy Mean rectum wall dose: 1.2 Gy					(Nassef *et al* 2016)
	Proton adaptive planning	Lung	CT to CT	Plastimatch (B-splines, demons), Velocity, Mirada, Raystation (Anaconda, Morfeus)	Not applicable	5 NSCLC patients with 9 repeated DIBH CTs	PTV-V95 decrease without adaptation by 14% (range: 1.5% − 40.5%) DIR-caused variations in PTV-V95 of accumulated doses on average 8.7% (range 1.0% − 26.3%)					(Nenoff *et al* 2021b)
	Proton adaptive planning	Head and neck	CT to CT	Plastimatch (B-splines, demons), Velocity,	Not applicable	1 Head and neck patient with 8 repeated CTs	After individually warping the dose with the different DIR algorithms, the volume for which the dose uncertainty in the accumulated dose was larger than 10% was (V_dosediff>10%_): Contralateral parotid: 28.1% Ipsilateral parotid: 13.9% Contralateral Retina: 9.4% Contralateral Macula: 8.9%					(Amstutz *et al* 2021a)
	Proton, Photon and Combined proton-photon adaptive planning	Lung	CT to CT	Plastimatch (B-splines, demons), Velocity, Mirada, Raystation (Anaconda, Morfeus)	Not applicable	5 NSCLC patients with 3 repeated DIBH CTs	Difference between the deposited fractional energy and the energy in the representation of the warped dose on the planning CT: Energy conservation violation in the accumulated energy averaged over treatment modalities and DIR algorithms compared to fractional deposited energy: GTV: 40.9% PTV: 32.1% OARs: randomly distributed within ±10% Energy conservation violation in traditional intensity-based DIR is linearly correlated to mass/volume variations.					(Wu *et al* 2023)

Also for proton therapy the dosimetric impact of using propagated structures for proton dose evaluation and optimization has been investigated: Qiao *et al* and Elmahdy *et al* investigated prostate structures propagated from CT to CT with the open source DIRs in Elastix (Elmahdy *et al* 2019, Qiao *et al* 2019). They gave an extensive geometrical evaluation (included in table 2) and dosimetric evaluation (included in table 3) that showed that DIR propagated structures can be used for optimization in online-adaptive intensity-modulated proton therapy (IMPT). Similar conclusions were found for lung cancer patients by Nenoff *et al*, showing that daily IMPT optimization on CT based on propagated, uncorrected structures was better than no adaptation (Nenoff *et al* 2021b). Daily manual recontouring on each CT gives a small additional benefit for some patients and OARs. They also investigated if including the inter-algorithm variation between structures propagated with DIR in the adaptive IMPT optimization could improve the adapted plan against structure uncertainties (Nenoff *et al* 2022). They found that adaptation on propagated, uncorrected structures showed a benefit over no adaptation for MRI-to-MRI registrations for pancreas and liver patients and CT-to-CT registrations for HN patients. Only for the HN patients including structure propagation uncertainties in the optimization significantly improved the adapted plan. Recently, Smolders *et al* compared the effect of different auto-segmentation methods, among those DIR based structure propagation, for the dosimetric quality of online adaptive proton therapy plans. They found the dosimetric influence of using automatic contours for the optimization to be small for OARs and larger for targets, with DIR propagated structures performing best for both OARs and targets (Smolders *et al* 2023a).

### 5.3. Mapped/Accumulated doses

In this section we outline the influence of DIR uncertainty on dose mapping and accumulation. For more details about dose mapping and accumulation, including direct dose mapping versus energy/mass mapping, biological considerations and (dis)appearing tissue please refer to the recent review of (Murr *et al* 2023).

#### 5.3.1. Intrafraction applications

A commonly proposed use of dose accumulation is for 4D treatment planning or the dose reconstruction of the dose in a moving area. Both, 4D optimisation (Graeff *et al* 2013, Engwall *et al* 2018, Spautz *et al* 2023) and 4D dose evaluation (Zhang *et al* 2019, Meijers *et al* 2020) require DIR and therefore show DIR-related uncertainties. Both are mostly applied in anatomical areas affected by breathing motion, registering all phases of a 4D CT or 4D MRI scan into a reference phase or average image (Rosu and Hugo 2012, Engwall *et al* 2018, Meijers *et al* 2020). Most 4D dose optimisation and dose calculation studies do not investigate DIR-facilitated dosimetric uncertainties (table 3). Those who do, report different metrics between different studies, to quantify these uncertainties. For example, (Ribeiro *et al* 2018) found differences in the target V95% of up to 11.34% for 4D dose accumulation of liver cancer proton therapy. In contrast, (Sarudis *et al* 2019) found only dose deviations of ≤3.0% between different visually acceptable DIRs in 4D lung volumetric modulated arc therapy (VMAT) dose accumulations. Mogadas *et al* tested five open-source registration algorithms on lung and liver SBRT, using the delta D95% of the target using 4D dose reconstruction compared to the static plan. For lung metastases, accumulated dose distributions were similar regardless of the DIR algorithm. In contrast, for liver metastases, accumulated dose distributions strongly varied, due to large DIR uncertainties in low contrast regions (Mogadas *et al* 2018).

#### 5.3.2. Interfraction applications

Another DIR-facilitated application has been to map doses re-calculated (or re-optimized) on 3D images from different fractions on the planning CT, to get an estimation of the total delivered treatment dose (Chetty and Rosu-Bubulac 2019, Ziegler *et al* 2019, Nenoff *et al* 2020). This technique has been extended to evaluate the validity of treatment plans with reduced margins (Wu *et al* 2009, van Kranen *et al* 2016, Lowther *et al* 2020b, van der Bijl *et al* 2022). The reporting of the uncertainties is very application-dependent and not standardised, which makes direct comparisons challenging (table 3). Examples of the magnitude of dose mapping uncertainties for interfractional changes include (Nenoff *et al* 2020), reporting differences caused by DIR uncertainties of 8.7% in the CTV of accumulated proton doses and (Wang *et al* 2018) reporting a maximum dose variation of 3.3 Gy for hollow organs in the abdomen for interfraction dose mapping. More recently, (Huesa-Berral *et al* 2022) reported a dosimetric uncertainty between fractions below 2 Gy in tumour and OAR in lung SBRT. This study also concluded that inter-fraction variations dominated and that dose accumulation for these patients should prioritise day-to-day changes over respiratory motion.

There have been several propositions to also predict uncertainties on the geometrical and the dosimetric level. The inter-algorithm variability was proposed to be used for geometric as well as dosimetric DIR uncertainties (Nenoff *et al* 2020, Amstutz *et al* 2021b). Probabilistic unsupervised DL methods have also been proposed to predict the variance of DVFs in interfraction datasets (Gong *et al* 2022, Smolders *et al* 2022b, 2022a).

#### 5.3.3. Intertreatment applications

Dose mapping and accumulation have been used in work on treatment method combinations and patient re-irradiation. Application of the technology presents the possibility of greater outcome modelling in combined methodologies, and long term outcomes in re-irradiation. Research regarding the combination of external beam RT and brachytherapy was done for cervical cancer patients (Vásquez Osorio *et al* 2015, Swamidas *et al* 2020, Zeng *et al* 2020). Van Heerden did not find clinically relevant improvements when using DIR for dose accumulation compared to adding uniform external beam RT doses or overlapping high dose volumes (van Heerden *et al* 2017).

In recent years, improved survival has led to an increase in the numbers of re-irradiations (Nieder *et al* 2013, Andratschke *et al* 2022) with particular focus made on cancers of the brain (glioma), lung, HN, abdomen, pelvis and spine (Abusaris *et al* 2012, Mantel *et al* 2013, De Ruysscher *et al* 2014, Nieder *et al* 2016). Dose from previous treatments can be deformed to the current anatomy to evaluate potential dose overlap (Meijneke *et al* 2013, Nix *et al* 2022). Thereby, being used to define safe dose tolerances in those previously treated regions (Embring *et al* 2021, Andratschke *et al* 2022, Brooks *et al* 2022, Nix *et al* 2022). In addition, DIR-facilitated dose warping can be used to correlate places of local failure with previously planned and/or delivered dose distributions (Boman *et al* 2017, McVicar *et al* 2018, Skjøtskift *et al* 2018, Embring *et al* 2021, Nix *et al* 2022). Registration algorithms are challenged by dramatic anatomical changes caused by the time between treatments, often months or years, not to mention sequels of treatments such as fibrosis resulting from radiation or surgery (Nix *et al* 2022, Vasquez Osorio *et al* 2023b). Systematic studies about the DIR uncertainties in the re-irradiation setting are rare, but some reports indicate that DIR uncertainty increases with the magnitude of anatomical changes, in particular for lung radiographic changes after SBRT (Mahon *et al* 2020). DIR uncertainty is only one of multiple uncertainty factors which makes the definition of organ constraints for re-irradiation challenging. The lack of standardised toxicity scoring or cumulative DVHs over multiple treatments, partially influenced by DIR uncertainty remain reasons why the recovery of organs over time is not well quantified. The calculation of biologically effective dose can improve the understanding of normal tissue responses over time (Brooks *et al* 2022, Nix *et al* 2022) and allow a better estimation of safe dose constraints during re-irradiation.

### 5.4. Other DIR-facilitated applications

DIR uncertainties can affect other medical physics and imaging applications.

#### 5.4.1. TCP and NTCP calculation

Currently, tumour control probability (TCP) and normal tissue complication probability (NTCP) models are built on planned doses. They are however designated to correlate to delivered doses which can differ from the planned dose. Dose accumulation of reconstructed doses on repeated images, requiring DIR in most anatomical areas, is the closest surrogate to the delivered dose that is available. The impact of DIR uncertainty on the accumulated doses directly affects the outcome calculation (Nenoff *et al* 2021a, Smolders *et al* 2023b). Deformation-free methods (Niemierko 1997, Niebuhr *et al* 2021) have their own (not well quantified) uncertainties. Niebuhr *et al* found larger differences when assuming a registration error of 3 mm, compared to changing alpha-beta values for prostate RT. (Niebuhr *et al* 2021) There is more research needed to fully understand and quantify the impact of DIR uncertainty for outcome calculation.

#### 5.4.2. Outcome modelling based on spatial/voxel-based analyses

**Figure 6.**
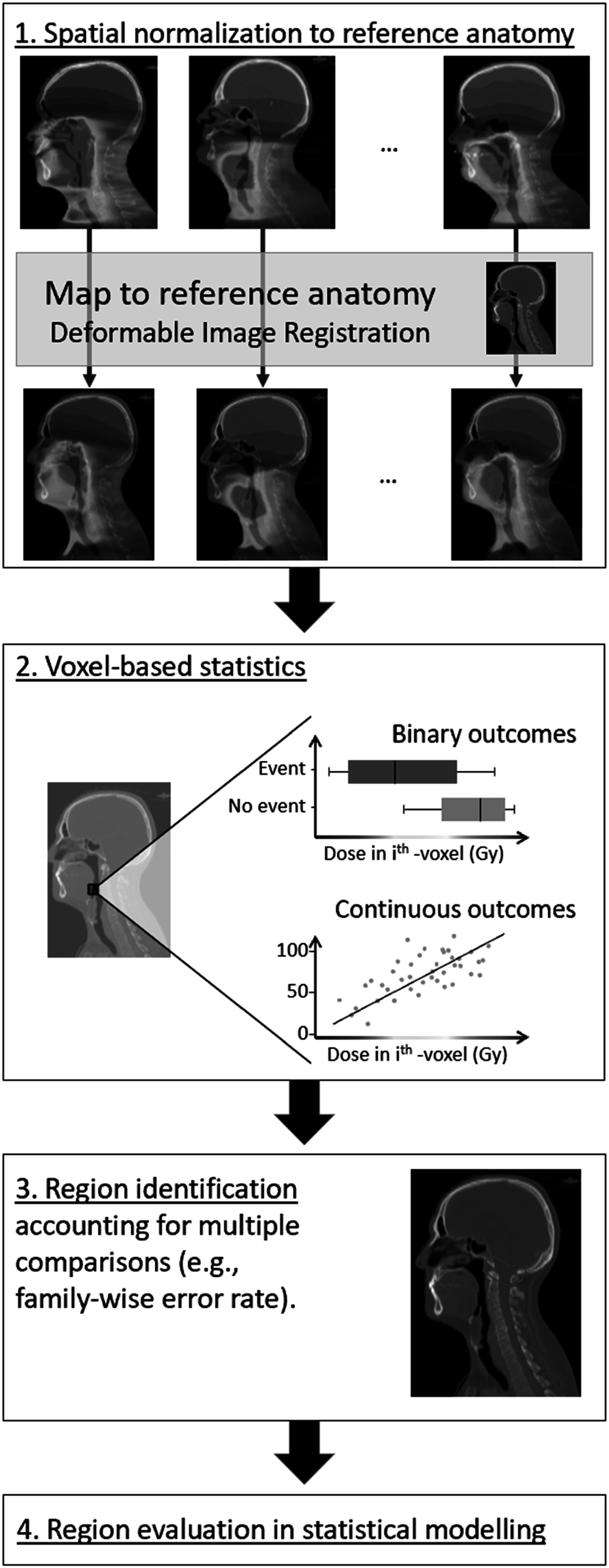
Voxel-based analysis applied to exploring local relationship between dose and a given outcome. This technique relies on deformable image registration to map the dose distributions of the studied patients to a selected reference anatomy.

Conventional outcome modelling simplifies the planned dose distribution to a single value, often using DVH statistics. Voxel-based analysis techniques that maintain the spatial distribution of doses have been used to explore local correlations between dose and treatment outcomes. Voxel-based analysis (figure 6) relies on DIR to ‘spatially normalise’ dose distributions into a common reference anatomy (Palma *et al* 2020, Shortall *et al* 2021). In summary, DIR is first performed between the planning CTs of each patient and an arbitrarily selected reference CT scan. The DIR result is then used to map the dose distributions to the reference anatomy allowing the local dose to be correlated with the studied outcome. The region is evaluated with statistical modelling, often quantifying the improvement in model discrimination when the dose to the identified region is included in a multivariable predictive model (including other demographic and clinical variables). The region is then used to generate hypotheses which are then tested and validated in external cohorts aiming at generating dose constraints to ultimately improve treatment outcomes.

With voxel-based techniques, doses to anatomical subregions have been linked to outcomes, such as the dose to the base of the heart to overall survival in lung RT (McWilliam *et al* 2017, Green *et al* 2020), the inferior–anterior hemi-anorectum dose to rectal bleeding in prostate RT (Dréan *et al* 2016) and the cricopharyngeus muscle, cervical oesophagus and the base of the brainstem dose to dysphagia in HN RT (Monti *et al* 2017).

Several measures to evaluate DIR uncertainty for voxel-based analysis have been proposed (Palma *et al* 2020, Shortall *et al* 2021, McWilliam *et al* 2023). Quantified DIR uncertainties are often incorporated in the analysis by treating them as random errors and blurring the mapped dose distributions (McWilliam *et al* 2017, Beasley *et al* 2018, Green *et al* 2020, Vasquez Osorio *et al* 2023a). Therefore, DIR uncertainties can result in a decrease of significance for small radiosensitive regions and local changes in their shapes.

## 6. Uncertainty tolerances of DIR-facilitated dosimetric procedures

**Figure 7.**
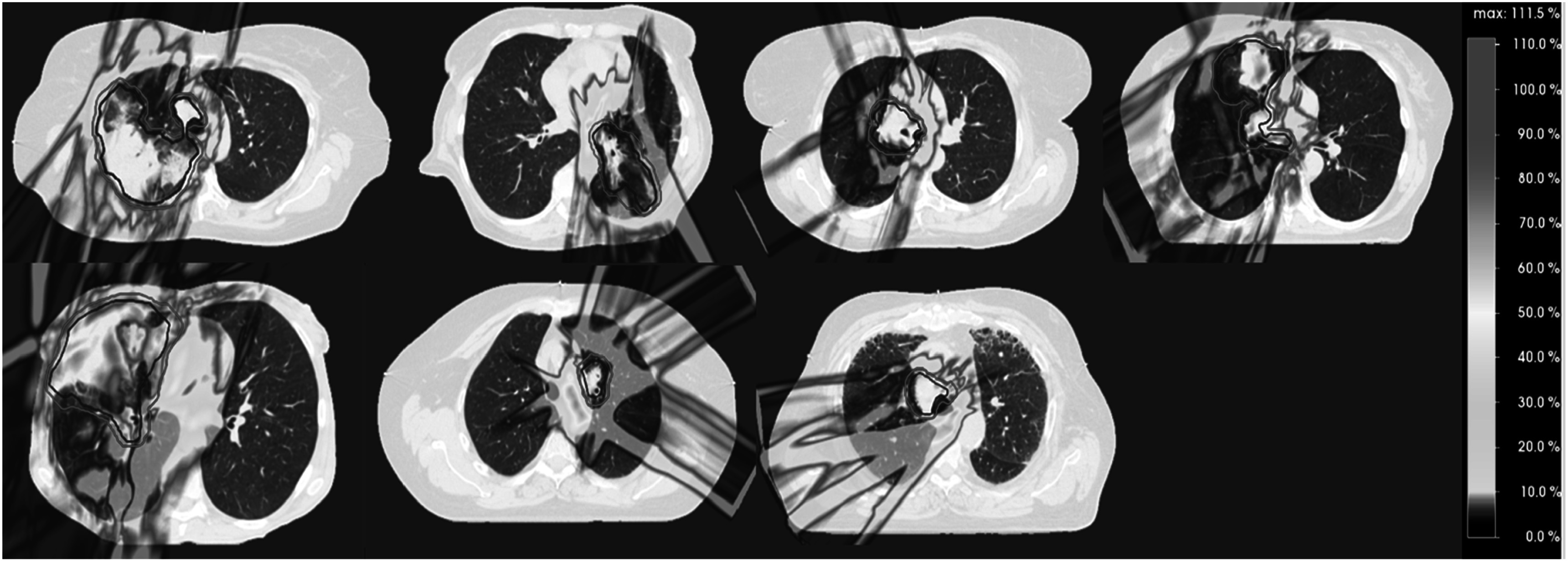
Examples of dose accumulation uncertainty, calculated as the voxel-wise difference between the maximum and minimum dose accumulated with one of six deformable image registration algorithms. Figure from (Nenoff *et al* 2020) with permission.

Specifying tolerances for the uncertainty in DIR-facilitated procedures is a challenging task and these should be based on clinical needs rather than achievable results. The demands on the accuracy of DIR vary by application. In a retrospective analysis, larger tolerances might be sufficient, while for interventional applications tighter tolerances might be indicated. For example, visualising a voxel-wise dose uncertainty map might be sufficient for a crude estimation of the dose in a re-irradiation case while precise DVH metrics along with their uncertainty estimation are necessary for correlating the dose to organs with outcome and toxicity data in clinical trials. In contrast to tolerances for geometric uncertainties, there is a scarcity of literature describing these for dose mapping or accumulation. There is no generally accepted approach on how to analyse and report DIR-related dosimetric uncertainties. Publications evaluating DIR-facilitated dosimetric differences are summarised in chapter 5 and table 3. A common finding in dose accumulation studies is that areas with steep dose gradients are more sensitive to DIR-facilitated uncertainties (Saleh-Sayah *et al* 2011, Swamidas *et al* 2020, Amstutz *et al* 2021b). Therefore, in areas with steep dose gradients DIR uncertainties are more relevant than in homogeneous dose areas or areas with low doses. Table 4 shows clinically relevant examples of dose gradients as well as geometric DIR uncertainties. Multiplying the dose gradient with the geometric DIR uncertainty gives an assessment of the dosimetric uncertainties expected in these situations. Low dose gradients are typically found in the central region of the target. Medium dose gradients are found in OARs in the beam path and high dose gradients are found close to the target boundary. As both the dose gradient and DIR uncertainty typically vary within an organ, voxel-wise dose uncertainty maps can visualise dose distribution uncertainties (figure 7).

**Table 4.** Voxel-wise dosimetric uncertainty as a function of the dose gradient and the uncertainty of the DIR. DIR: deformable image registration.

	Dose gradient		
	Low	Medium	High
DIR uncertainty	1 %/mm	10 %/mm	25 %/mm
Low 1 mm	1%	10%	25%
Medium 5 mm	5%	50%	125%
High 10 mm	10%	100%	250%

**Figure 8.**
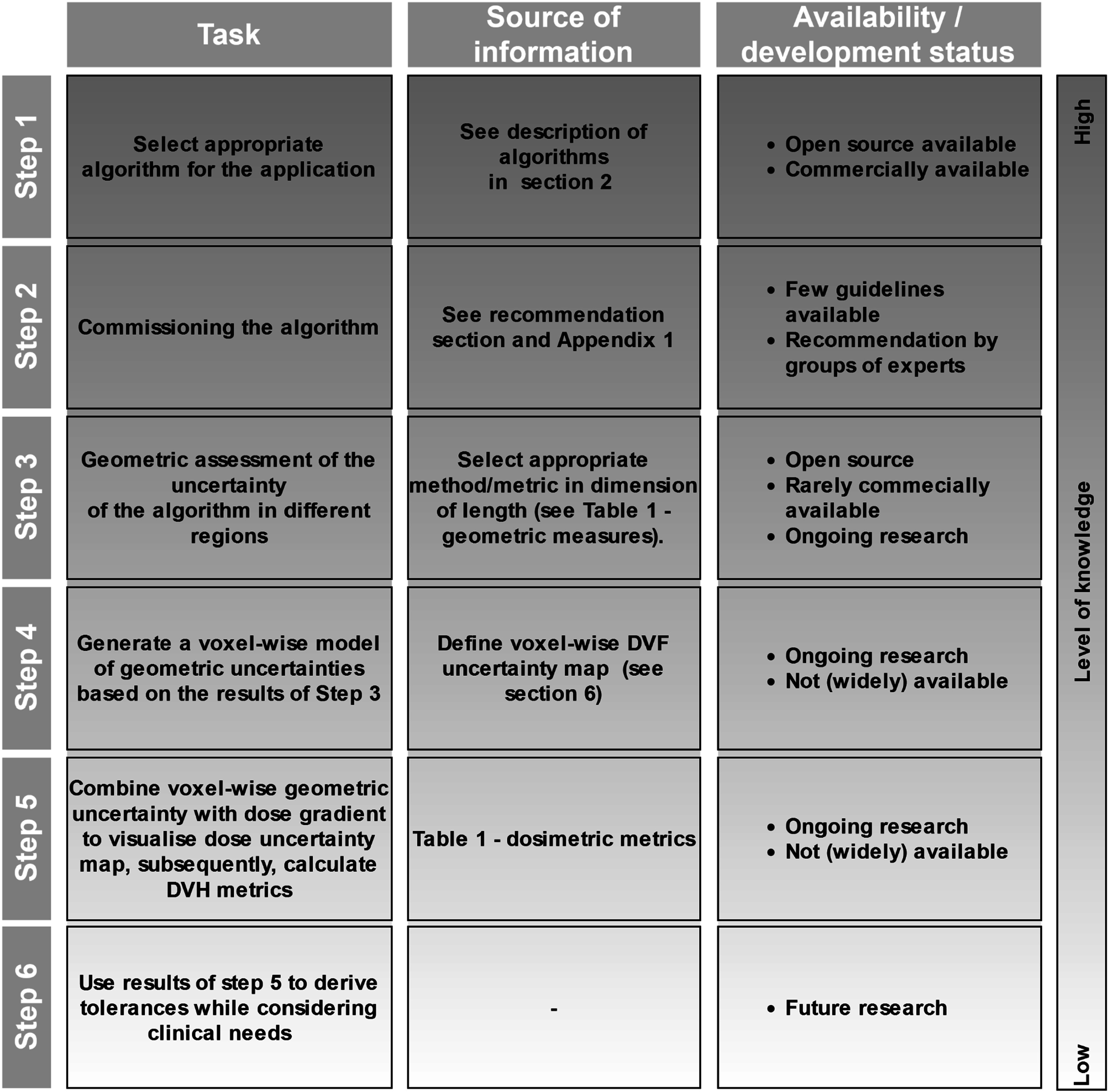
An example approach on how to assess dosimetric uncertainties of accumulated dose caused by DIR-uncertainties. The shading indicates the level of knowledge/confidence of the individual steps.

Since there is no standard agreed upon in the literature on how to quantify dosimetric DIR uncertainties or tolerances, we propose a short ‘recipe’ (figure 8). The first step is the selection of the DIR algorithm. Second, the algorithm must be commissioned for the specified application (recommendations in chapter 7 and commissioning document in the supplement). Third, the DIR uncertainty is evaluated using geometric measures. We consider geometric measures in dimension of distance (e.g. target registration error (TRE), MHD) necessary to define tolerances. The quantification of geometric measures needs to be done for different structures and points of interest such as targets, OARs, anatomical landmarks close to the target or in the beam path.

Steps 1–3 are described in multiple recommendations (Brock *et al* 2017, Barber *et al* 2020, Lowther *et al* 2022). In step 4 a voxel-wise geometric uncertainty map of geometrical measures is created (Amstutz *et al* 2021b). The simplest method is using the worst-case or average difference distance in all directions for all voxels of a given region or structure. More individualised methods have been investigated (Amstutz *et al* 2021b, Smolders *et al* 2022b, 2023c) and provide patient specific voxel-wise uncertainties maps. We recommend using such voxel-wise uncertainty maps whenever possible. However, due to the lack of commercial implementations, simpler global geometrical metrics are easy-to-implement alternatives. Using these metrics may lead to locally over- or underestimated geometric uncertainties, but its use is an improvement over no geometric uncertainty estimation, and will help pave the way to include such concepts in clinics.

In step 5 the geometric uncertainty map is applied to the dose by calculating the scalar product of the dose gradient and the geometric uncertainty of the DIR transformation on a voxel-wise level. This uncertainty map can be used to calculate DIR-facilitated variations of DVH parameters or DVH bands. To define geometric tolerances of DVFs, steps 6 to 3 can be propagated backwards: starting with a maximum allowed DVH variation or uncertainty in a given voxel resulting in a maximum allowed DVF uncertainty. Since multiple relevant methods are not yet widely available or still require future research the definition of tolerances is not trivial.

Another method to calculate the required accuracy of a registration to achieve a given tolerance is the distance-to-dose difference (DTD), proposed by Saleh-Sayah *et al* The DTD indicates how large local registration errors can be before they introduce mapping errors breaching the given tolerance. For example accurate DVFs (1 mm) are required in high dose gradient regions while large DVF errors (>20 mm) are acceptable in low dose gradient regions. Another approach is to divide the acceptable dosimetric tolerance by the dose gradient (TDG). Compared to the TDG, the DTD gives a more conservative assessment (Saleh-Sayah *et al* 2011, Saleh *et al* 2014).

## 7. Recommendations

Several publications have offered recommendations for methods and action thresholds for assessing registration quality. We endorse these efforts. This section summarises these recommendations and extends recommendations for the community.

### 7.1. Recommendations for patient-specific use

TG-132 recommends visual inspection for patient-specific use, using split-screen, fusion, contour overlay, or other tools (Brock *et al* 2017). Visualisation should focus on alignment of anatomic landmarks, organ or tissue boundaries, vessels, and other distinct features. When software allows, the displacement field should be inspected to identify implausible deformations. Qualitative assessment can optionally be verified using quantitative metrics such as those summarised in table 2. TG-132 also recommends a threshold of 2–3 mm accuracy in TRE and MDA, although this is not achievable in practice (Rong *et al* 2021). Vector field smoothness should be tested for locations with negative Jacobian determinant. We suggest this threshold might lie between 0.2 and 2.0. MIRSIG recommends additional tests on the displacement field using DVF histograms, transitivity errors, and harmonic energy, but no thresholds are provided. TG-132 recommends a 2–3 mm threshold for inverse consistency, and a 0.8–0.9 threshold for DSC, with the caveat that DSC varies widely by structure volume.

Applications using dose deformation or dose accumulation should focus on the important regions of interest. Usually these are the volumes with meaningful dose levels, relevant structures and high dose gradients. The recipe proposed in chapter 6 can provide guidance how to calculate dosimetric uncertainties on a voxel-wise level.

### 7.2. Recommendations for commissioning

System commissioning requires testing software interchange, and TG-132 recommends using a physical phantom for this purpose. It also recommends testing on digital phantoms to recover known, artificial deformations. Best practices prospectively evaluate registration software on treatment sites of interest, but there are few guidelines on this. Glide-Hurst *et al* recommend centralised review of each fraction for at least the first three cases in adaptive therapy clinical trials (Glide-Hurst *et al* 2021). We recommend five representative patient cases to assess with quantitative metrics. These metrics should be compared to typical values from the literature (tables 2 and 3, commissioning document in the supplement) and with inter-observer variability.

### 7.3. Recommendations for developers, vendors, and the community

TG-132 recommends that vendors provide a basic description of the registration algorithm, vector field export, and basic quantitative tools (DSC, MDA, TRE). Unfortunately software providers still fail to apply these quantitative assessment tools (Rong *et al* 2021). More recently, Murr *et al* evaluated contour distance metrics and DVF analysis tools, such as DVF visualisation, transitivity analysis, and Jacobian determinant (Murr *et al* 2023). They recommend vendors to implement dose uncertainty tools, a region of interest (ROI) tool to limit registration domain, multiple algorithms for sensitivity analysis, and a greater selection of state-of-the-art algorithms.

In additional to these recommendations, we add:

• Tools for generating artificial warps
• ROI tools for quantitative metrics within a contour or dose level
• DIR correction tools, such as a smudge tool to locally push the registration, vector field smoothing tool, landmark-based correction, and contour-based correction
• Open access resources of reference images, structures, landmarks, and vector fields
• Tools to restrict DIR to be locally rigid or locally mass-preserving
• Tools to import and export DVFs in a consistent dicom format
• Voxel-wise uncertainty quantification and visualisation

### 7.4. Recommendations for future research

Finally, we propose areas where research is still needed.

**TCP and NTCP metrics.** It is unclear how DIR-generated dose distributions are related to clinical outcomes, considering uncertainties. Uncertainties in DIR-generated doses should be quantified with the metrics described in table 1 and utilised with the aim of generating more accurate TCP and NTCP models.

**DIR failure modes.** While it is possible to obtain typical uncertainty estimates during commissioning, many DIR algorithms have unexpected failure modes which are hard to enumerate. It is desirable to better understand the causes of these failures so that automated tests can be performed.

**Uncertainty estimation methodology.** There are multiple methods in use for estimating the uncertainty of DIR, and they are difficult to compare as they measure different aspects. Efforts should be made to find consensus on which methods should be preferred for each application.

**Avoiding DIR.** For online ART, improvements in imaging and dose calculation could eliminate the need to deform images with DIR for daily dose calculation and plan optimisation, and thereby eliminate it as a source of overall uncertainty. To evaluate the total accumulated treatment dose, DIR will remain necessary.

## 8. Summary

DIR is a powerful and versatile tool for RT. It has many applications, but is also associated with considerable uncertainties. Many clinical DIR solutions have been implemented, but they generally lack tools for uncertainty quantification. In the community, there are no agreed thresholds to distinguish between a good or bad DIR result when using a combination of geometric and dosimetric measures. Multiple quantification metrics, mostly using geometrical measures, and tolerances have been proposed. The reporting of dosimetric measures and uncertainties caused by DIR uncertainty is less standardised and highly application dependent. It is important to reach an agreement and standardisation in the evaluation of DIR uncertainties for different RT applications. In this review we summarised DIR-facilitated uncertainties for different applications and gave recommendations on the quantification of DIR uncertainties. We then outlined a potential path towards definition of tolerances. It should be emphasised that the presented recommendations are only a starting point, they should be challenged and refined by the community.

## Data Availability

The paper is a review paper, no new data was acquired. The data that support the findings of this study are available upon reasonable request from the authors.

## Funding

LN: reports funding from the Swiss National Science Foundation (SNSF): grant 199943 and from the NIH R01 CA229178. FA: reports funding from the Swiss Cancer Research Foundation (KFS-4528-08-2018) and Krebsliga Schweiz Research Grant (KFS-4517-08-2018). MM: reports funding from DFG ZI 736/2-1 and MU 6403/1-1 (PAK 997/1-1). MH: supported by the National Measurement System of the UK’s Department for Business, Energy and Industrial Strategy. GCS: reports funding from the NIH R01 CA229178. EVO: reports funding by Cancer Research UK RadNet Manchester [C1994/A28701]. BAH, MF, WL, YZ: No funding to disclose.

## Conflicts of interest

LN: No conflict of interest. FA: No conflict of interest. MM: reports institutional research agreements with Elekta, Philips, TheraPanacea, Kaiku, PTW Freiburg. BAH: No conflict of interest. MF: No conflict of interest. MH: reports institutional collaboration agreement with RaySearch. WL: reports institutional research agreements with Brainlab, Elekta, Philips and RaySearch. YZ: No conflict of interest. GCS: No conflict of interest. EVO: No conflict of interest.
